# Recent developments in the kinetics of ruptures of giant vesicles under constant tension

**DOI:** 10.1039/d1ra04647k

**Published:** 2021-09-02

**Authors:** Mohammad Abu Sayem Karal, Md. Kabir Ahamed, Marzuk Ahmed, Zaid Bin Mahbub

**Affiliations:** Department of Physics, Bangladesh University of Engineering and Technology Dhaka-1000 Bangladesh asayem221@phy.buet.ac.bd +880-2-58613046 +880-2-9665613; Department of Mathematics and Physics, North South University Dhaka-1229 Bangladesh

## Abstract

External tension in membranes plays a vital role in numerous physiological and physicochemical phenomena. In this review, recent developments in the constant electric- and mechanical-tension-induced rupture of giant unilamellar vesicles (GUVs) are considered. We summarize the results relating to the kinetics of GUV rupture as a function of membrane surface charge, ions in the bathing solution, lipid composition, cholesterol content in the membrane, and osmotic pressure. The mechanical stability and line tension of the membrane under these conditions are discussed. The membrane tension due to osmotic pressure and the critical tension of rupture for various membrane compositions are also discussed. The results and their analysis provide a biophysical description of the kinetics of rupture, along with insight into biological processes. Future directions and possible developments in this research area are included.

## Abstract 2

The kinetics of ruptures of giant vesicles under constant tension have been investigated under various conditions, which will help in the application of these techniques in the biomedical, bioengineering, and biotechnological fields.

## Graphical abstract

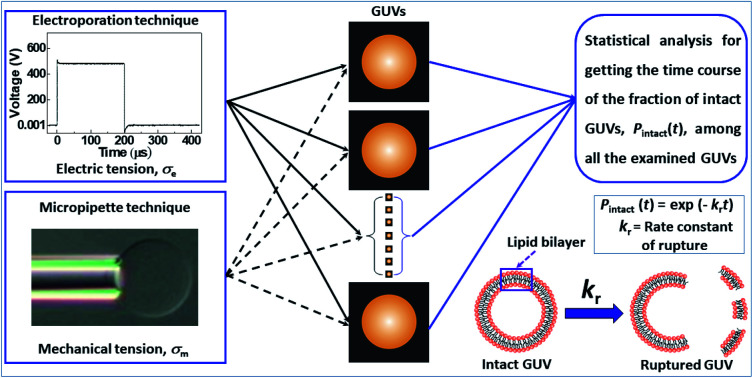

## 1. Introduction

Stretching plays a crucial role in the plasma membranes of prokaryotic and eukaryotic cells for the opening of mechanosensitive ion channels (MSCs).^1–3^ Stretching induces lateral tension in the membranes, and when this tension exceeds a critical value, rupture of vesicles and lysis of cells (cell death) occur.^4–6^ Essentially, vesicles with nano- to micrometer diameters are closed and spherical structures formed by a double layer of lipid. Other factors such as electric fields,^7,8^ osmotic pressure^9–11^ and the antimicrobial peptide Magainin 2 (ref. 12 and 13) also induce membrane tension leading to the rupture of vesicles. Externally applied mechanical tension in the membranes also plays an important role in the action of various types of peptides (*e.g.*, antimicrobial and cell-penetrating peptides) in lipid vesicles.^14^ Hence, study of the kinetics of the rupture of vesicles due to different kinds of tension (*e.g.*, electric and mechanical) is a matter of interest in the community.

Vesicles are central in a huge number of investigations, and are used as models of cells^15^ as well as for delivering drugs to specific body organs.^16–19^ Among the various sizes of vesicles, giant unilamellar vesicles (GUVs) with diameters equal to or greater than 10 μm have been used in various experiments,^20,21^ since they provide the opportunity to observe a ‘single GUV’ and the corresponding interactions with the membrane-active agents using optical microscopy.^22,23^ The mechanical properties of membranes can be obtained by measuring the area compressibility modulus of GUVs. The critical tension for the rupture of GUVs is used to evaluate the strength of membranes.^24,25^

The technique of applying electric tension to membranes to rupture GUVs *via* irreversible electroporation (IRE) has been utilized to study the ablation of tumor and cancer cells.^26–30^ Usually, IRE induces lateral electric tension in the membranes of GUVs, which can be calculated by the Maxwell stress tensor.^31–34^ In the IRE technique, GUVs are permanently permeabilized by applying micro- to millisecond electric pulses. Recently, a technique has been developed for applying a constant electric tension to the membranes of GUVs using IRE, by which the electrodeformation, electrofusion, and rupture of charged and cholesterol-containing membranes have been investigated.^8,35–37^ In addition, the effect of osmotic pressure on the constant electric-tension-induced rupture of GUVs has also been studied.^11^

Mechanical tension is applied to the membranes of GUVs using the micropipette aspiration technique, which helps in understanding the processes of pore formation and vesicle rupture and in estimating the elasticity of membranes.^38^ In dynamic tension spectroscopy, mechanical tension is applied at various increasing rates to analyze the rupture mechanism of the corresponding GUV.^5^ As the rate of applied tension increases, the time of pore formation decreases, and hence faster stretching, up to the same constant magnitude, enhances the kinetics of rupture. Recently, an experimental technique has been developed for applying a constant mechanical tension to the membranes and investigating the rupture of GUVs.^39^

Generally, rupture occurs in GUVs due to the formation of pores in the membranes. Studies of such pore formation are used in various biomedical applications.^1,40–43^ Hence, it is necessary to understand the mechanism of pore initiation, formation and closing. The model of continuous trajectories of pore formation describes the dynamics of poration in a lipid bilayer under electrical and mechanical stresses.^44^ The life cycle model of pores describes pore opening, construction and closing.^45^ Molecular dynamics (MD) simulations have shown a linear dependence of the energy required for pore formation on the applied field.^46^ Simulation works dealing with molecular transport through a single nanopore have indicated that the rate of molecular transport depends on the size of the fluorescent probe and also the size of the GUVs.^47^ It is worth mentioning that a single pore can also be formed in a GUV *via* intense optical illumination, and it is possible to analyze the pore closure dynamics including line tension.^4,48^ In the lipid bilayer, the rate of closure of pores mainly depends on the line tension (or line free energy per unit length at the pore edge).^49^ Subsequently, various experiments and theories have been used for analyzing pore closure dynamics, in which larger pores are formed by stretching.^50–52^

To understand the mechanism of tension-induced rupture of GUVs, the kinetics of rupture have been investigated in various studies^38,46,53,54^ in which constant tension was not considered. Additionally, the kinetics (*i.e.*, rate constants) of rupture have been extensively studied using constant electric and mechanical tension.^39,55^ In this review, recent developments in the kinetics of rupture of GUVs induced by constant electric or mechanical tension are presented. The IRE technique induces constant electric tension in the membranes of vesicles, whereas constant mechanical tension is induced by the micropipette aspiration technique. We describe how the membrane composition, bathing medium, cholesterol content in the membrane, lipid composition and osmotic pressure affect the rate constant of rupture in GUVs along with the membrane stability. The physical properties of membranes, such as line tension under various conditions, are discussed. The membrane tension due to osmotic pressure and the critical tension of rupture are also presented.

## 2. Materials and methods

This section gives brief descriptions of the production of GUVs and the methods for the constant-electric-tension- and constant-mechanical-tension-induced rupture of GUVs. The necessary illustrations are provided for clear understanding of the calculation of kinetics, *i.e.*, the rate constant of rupture.

### 2.1. Synthesis of GUVs

There are various methods for the preparation of GUVs.^56–58^ Among them, the well-known natural swelling method for the synthesis of GUVs was developed in 1961.^59^ Since then, this simple but effective method has been used for preparing GUVs.^10,23,38,60,61^ For the production of GUVs, 1,2-dioleoyl-*sn*-glycero-3-phospho-(1′-*rac*-glycerol) (sodium salt) (DOPG), 1,2-dioleoyl-*sn*-glycero-3-phosphocholine (DOPC), 1,2-dilauroyl-*sn*-glycero-3-phospho-(1′-*rac*-glycerol) (sodium salt) (DLPG), and 1,2-ditridecanoyl-*sn*-glycero-3-phosphocholine (DTPC) were purchased from Avanti Polar Lipids Inc. (Alabaster, AL). Bovine serum albumin (BSA), 1,4-piperazinediethanesulfonic acid (PIPES), ethylene glycol-bis(2-aminoethylether)-*N*,*N*,*N*′,*N*′-tetraacetic acid (EGTA) and calcein were purchased from Sigma-Aldrich (Germany). Cholesterol (chol) was purchased from WAKO pharmaceuticals (Japan). The GUVs were prepared in PIPES buffer (10 mM PIPES, pH 7.0, 150 mM NaCl and 1 mM EGTA) using the natural swelling method^59,60^ Here, this method is described briefly. A mixture of 1 mM DOPG and DOPC (about 200 μL) was placed in a glass vial and dried using a gentle flow of nitrogen gas to produce a thin and homogeneous lipid film. By keeping the vial in a vacuum desiccator for 12 hours, the residual chloroform in the film was removed. To synthesize the cholesterol-containing membranes, a mixture of 1 mM DOPG, DOPC and cholesterol was used. Subsequently, 20 μL MilliQ water was added to the vial for pre-hydration at 45 °C for 8 minutes. After pre-hydration, the sample was incubated with 1 mL buffer containing 0.10 M sucrose for 3 hours at 37 °C. To prepare GUVs containing a water-soluble fluorescent probe (calcein), the vesicles were incubated with buffer and 0.10 M sucrose containing 1 mM calcein. The incubated GUV suspension (unpurified) was centrifuged at ∼13 000×*g* (here *g* is the acceleration due to gravity) for ∼20 minutes at ∼20 °C to remove the multilamellar vesicles (MLVs) and lipid aggregates, as these elements sedimented at the bottom of the Eppendorf tubes.^62–64^ After centrifugation, the supernatant was collected for purification. The unpurified GUV suspension was purified using the membrane filtering method.^65^ Hence, the inside and outside of the GUVs were buffer containing 0.10 M sucrose and 0.10 M glucose, respectively.

The size distribution of the unpurified and purified GUVs prepared using different conditions has been reported previously.^60,66^ The size range for the unpurified GUVs was 3.0–60 μm, and the number of smaller vesicles with sizes of less than 10 μm was large. On the other hand, the size range for the purified GUVs was 7.0–60 μm, and the number of smaller vesicles with sizes of less than 10 μm was very small. Hence, the average size of the purified GUVs was approximately 20 μm, whereas the average size of unpurified GUVs was approximately 11 μm.

### 2.2. Constant electric tension *via* the electroporation technique

It is well documented that electric fields induce the rupture of GUVs.^31–34,46,67,68^ As lipid membranes are impermeable to ions, the electric field (*E*) polarizes the internal and external free charges of the buffer of the GUVs. The accumulation of the free charges of the buffer increases the membrane potential (*V*_m_), resulting in stretching in the membranes. This stretching induces lateral membrane tension in the GUVs. The constant electric tension (*σ*_e_) induced by IRE is expressed as follows:^31,34^1
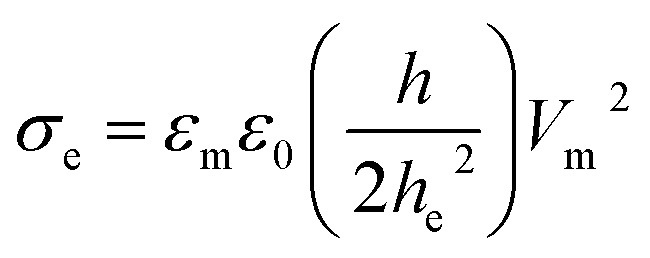
where *ε*_m_ is the permittivity of the membrane (∼4.5),^69,70^*ε*_0_ is the permittivity of free space, *h* is the membrane thickness (∼4 nm) and *h*_e_ is the dielectric thickness of the membrane (∼2.8 nm).^71^ It is possible to employ a simple model in order to consider the uniform field on the GUV with negligible membrane conductivity. The induced membrane potential, *V*_m_ = 1.5*RE* cos *θ*, where *θ* (= 0°) is the angle between the radius of GUV (*R*) (from the GUV centre to the evaluation point) and the electric field (*E*).^72^ For a GUV with *R* = 10 μm and *E* = 553 V cm^−1^, *V*_m_ = 0.83 V. After simplification of eqn (1), the constant electric tension can be expressed as follows:^37^

2 *σ*_e_ = 22.86*R*^2^*E*^2^ [mN m^−1^]

Eqn (2) shows that the tension can be kept constant by changing the electric field depending on the specific size of the GUVs. For example: *σ*_e_ = 5.0 mN m^−1^ for *R* = 10 μm and *E* = 468 V cm^−1^; *σ*_e_ = 5.0 mN m^−1^ for *R* = 8 μm and *E* = 585 V cm^−1^; *σ*_e_ = 7.0 mN m^−1^ for *R* = 10 μm and *E* = 553 V cm^−1^, *etc.*

**Fig. 1:**
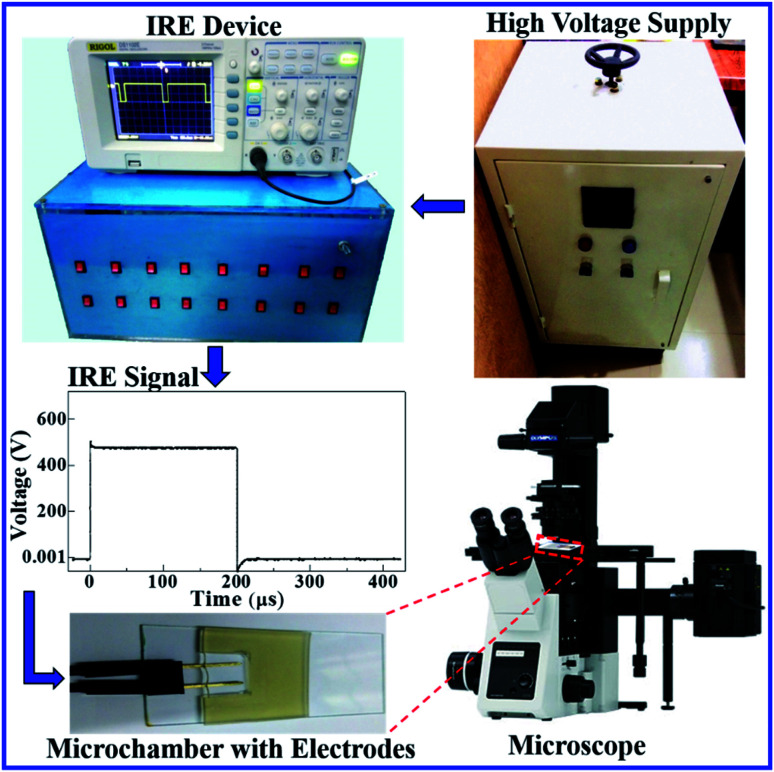
The experimental set-up for the electroporation technique.

Fig. 1 shows an ingeniously developed IRE technique. The detailed electronic circuit of the technique was published previously.^8,73^ The electroporation device mainly consists of an ultra-short pulse generator and a high-voltage power supply. These parts are connected to a switching circuit to obtain the final IRE signal with a microcontroller system. The DC square pulses are created by a multivibrator. A MOSFET (2SK3748, N-Channel Power MOSFET, ON Semiconductor, SCILLC)-based switching circuit was designed for generating square pulses.

To develop the high-voltage power supply, initially, a single-phase transformer (No. 40 KVA) is driven using 220 V AC, which provided 0–800 V AC with 5 A. The secondary voltage is converted using a full-wave rectifier to obtain the required DC voltage with proper regulation. The square pulses are generated at low-voltage by the pulse generator, and are used to switch the high-voltage power MOSFET to obtain the desired output. A microcontroller (ATMEGA8, 16PU, Atmel Corporation) is used to control the frequency, pulse width and number of pulses of the IRE signal accurately. The developed IRE device provides electric field pulses with a pulse width of 200 μs and a frequency of 1.1 kHz. The microcontroller-based IRE signal is applied to the GUVs (which are kept in a custom-built U-shaped microchamber) through a gold-coated electrode with a length of 17.0 mm and a width of 2.54 mm (SH-17P-25.5, Hellotronics).

In the electroporation technique, first, a ‘single GUV’ located between the electrodes is focused upon. The constant electric tension is applied and the rupture of that GUV is investigated. This is the first observation of a ‘single GUV’ in the first microchamber. Next, a similar experiment is done in the second microchamber for another ‘single GUV’ under the same electrical stress. This process continues for 20–25 microchambers for different ‘single GUVs’ under the same stress. In this way, the same tension (uniform stress) is applied to several ‘single GUVs’ in different microchambers.

**Fig. 2:**
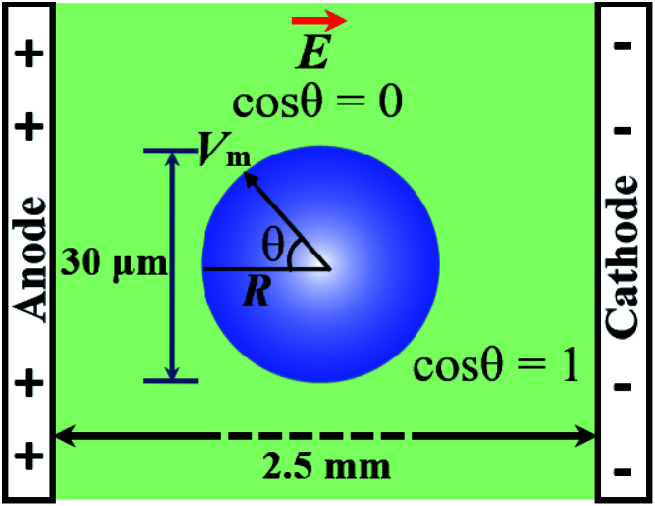
The experimental design of the IRE technique for pore formation in GUVs. The electric field induces the modification of the transmembrane potential of the GUV. *θ* is the angle between the radius of the GUV (*R*) (from the GUV center to the evaluation point) and the electric field.

To apply the constant electric tension to the membrane of the GUV, initially, the electric field (*E*) is kept at a value (*i.e.*, ∼150 V cm^−1^) that creates an electric tension of *σ*_e_ = ∼0.5 mN m^−1^, while the GUV remains between the electrodes (Fig. 2). Then, the electric field is increased quickly (∼8 s) to a specific value and is kept constant for a specific time (*i.e.*, 60 s). The starting time of the rupture of the GUV corresponds to the time of pore formation in the membranes, which has a time range of less than 1 s. The value of *σ*_e_ for the corresponding electric field is calculated using eqn (2).

### 2.3. Constant mechanical tension *via* the micropipette aspiration technique

A large number of studies have investigated the mechanical-tension-induced rupture of GUVs^5,24,55,74–76^ using the micropipette aspiration technique. The applied constant mechanical tension (*σ*_m_) on the membrane of the GUV can be expressed as a function of the suction pressure Δ*P*, which is the pressure difference between the outside (*P*_out_) and the inside of the micropipette (*P*_in_) (*i.e.*, Δ*P* = *P*_out_ − *P*_in_) as follows:3
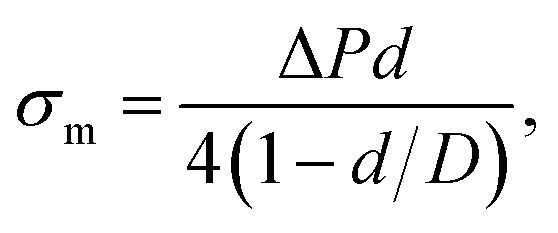
where *d* is the internal diameter of the micropipette and *D* is the diameter of the spherical part of the GUV exterior to the micropipette.

**Fig. 3:**
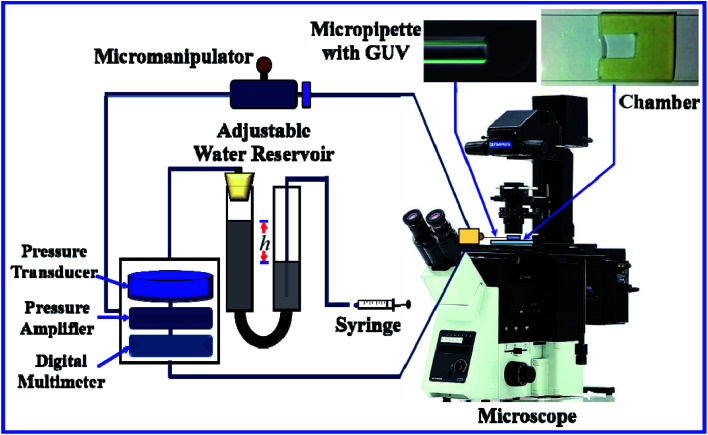
The experimental set-up for the micropipette aspiration technique.

Fig. 3 shows the experimental set-up of the micropipette aspiration technique, in which a suction pressure Δ*P* is applied to the micropipette using an adjustable water reservoir or a pump.^76^ The change in the area of the GUVs is determined microscopically using image analysis. The value of Δ*P* is measured using the difference in height (*h*) between the micropipette tip and the top of the reservoir, and hence, Δ*P* = *ρgh*, where *ρ* is the density of water and *g* is the acceleration due to gravity. If there is a flow, the pressure drops along the microcapillary, and hence, the equation would be Δ*P* = *ρgh*(1 − *U*/*U*_f_), where *U* is the velocity of the aspirated material and *U*_f_ is the velocity that it would have if flowing freely. Generally, *U*_f_ is several orders of magnitude higher than *U* (*U*_f_ ≈ 4 μm s^−1^ for a capillary with an internal radius *r* ≈ 5 μm), and hence, Δ*P* ≈ *ρgh*. To prevent strong adhesion of the membrane to the glass micropipette, which could lead to overestimation of the mechanical properties of the cell, bovine serum albumin is generally used.

**Fig. 4:**
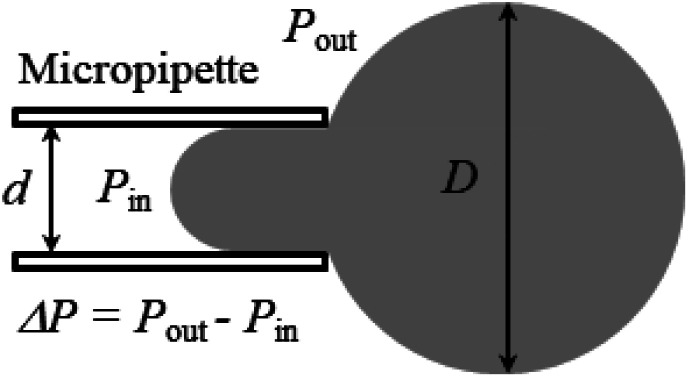
An illustration of the application of mechanical tension to the membrane of a GUV using the micropipette aspiration technique.

In this technique, at first, a ‘single GUV’ is held at the tip of the micropipette by applying a small suction pressure (equivalent tension of ∼0.5 mN m^−1^) and the GUV is aspirated to the targeted tension quickly (∼10 s), and then kept at this tension for a particular time, *e.g.*, 10 min. The GUV is observed until its complete aspiration into the micropipette occurs (Fig. 4). The GUV is ruptured due to pore formation in the membrane. The time of rupture corresponds to the time at which the GUV is aspirated, with a time range of less than 1 s. In this way, approximately 20–25 ‘single GUVs’ are investigated from a microchamber using the ‘single GUV’ method.^20^

### 2.4. Measurement of the rate constant of rupture of GUVs

**Fig. 5:**
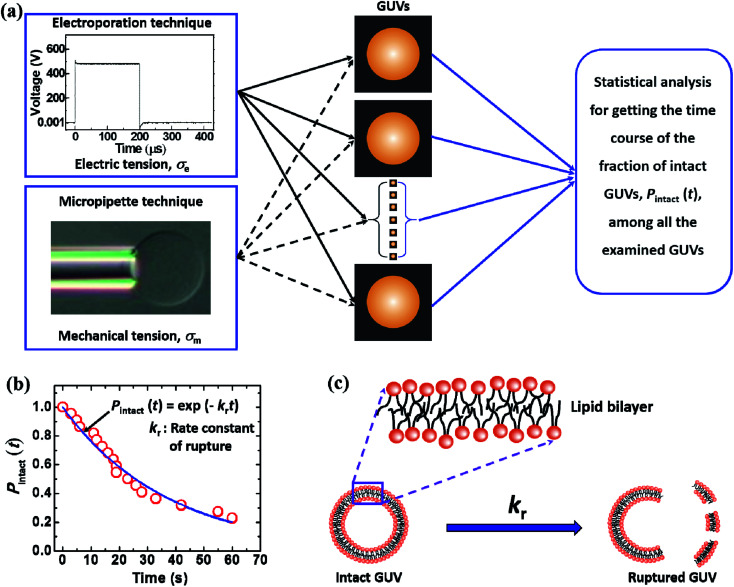
An illustration of the calculation of the rate constant of rupture of GUVs. (a) Electric tension or mechanical tension is applied to several ‘single GUVs’. (b) The calculation of the rate constant of rupture. (c) The transition from an intact GUV to a ruptured GUV.

Fig. 5 illustrates the calculation of the kinetic constant, namely, the rate constant of rupture of GUVs. First, a constant electric tension (*σ*_e_) or mechanical tension (*σ*_m_) is applied to a ‘single GUV’ and its structural change (whether the GUV is ruptured or not) is observed as a function of time using a phase contrast fluorescence microscope. Next, a similar experiment and observation are carried out for another ‘single GUV’ under the same constant tension. This procedure is then repeated for many ‘single GUVs’ (Fig. 5(a)). The time course of the fraction of intact GUVs, *P*_intact_ (*t*), out of all the examined GUVs is fitted with a single-exponential decay function as follows (Fig. 5(b)):

4 *P*_intact_(*t*) = exp(−*k*_r_*t*),

where *k*_r_ is the rate constant of the rupture of the GUVs for a specific constant tension and *t* is the time duration of the application of the constant tension to the membranes. The rate constant of rupture provides the rate of transition from the intact vesicle to the ruptured vesicle (Fig. 5(c)). The average value of *k*_r_ with standard deviation is calculated from several independent experiments under the same constant tension.

## 3. Results

### 3.1. Effects of surface charge density and salt concentration on the constant-electric-tension-induced rupture of GUVs

**Fig. 6:**
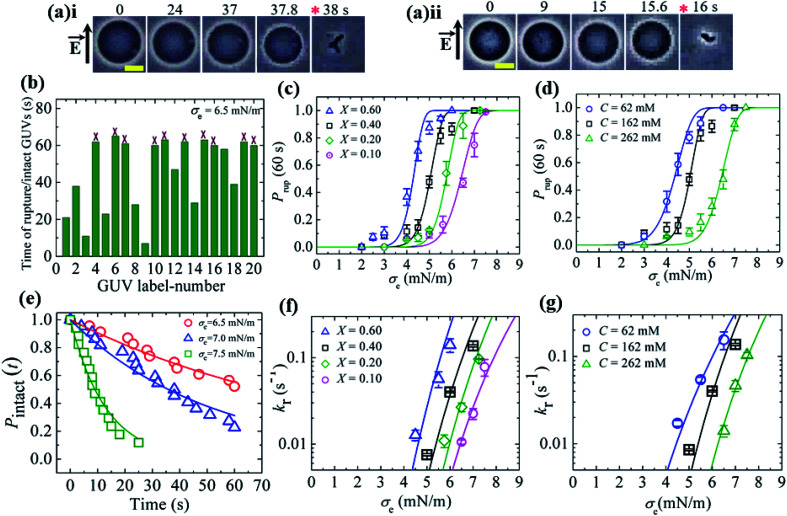
The constant-electric-tension (*σ*_e_)-induced rupture of GUVs under various conditions. (a) Phase contrast images of the rupture of (i) the first ‘10%DOPG/90%DOPC-GUV’ and (ii) the second ‘10%DOPG/90%DOPC-GUV’ at *σ*_e_ = 6.5 mN m^−1^. The left-side arrow indicates the electric field direction. The numbers above the images indicate the time (in seconds) after applying *σ*_e_. The scale bar corresponds to a length of 15 μm. The time of rupture is indicated by an asterisk (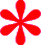). (b) The time of stochastic rupture and intact status of several ‘single 10%DOPG/90%DOPC-GUVs’ (*n* = 20) at *σ*_e_ = 6.5 mN m^−1^. The crosses (×) above the bars indicate that the GUV was intact at 60 s. (c) The *σ*_e_-dependent probability of rupture before 60 s, *P*_rup_ (60 s), for *X* = 0.60 (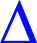), 0.40 (□), 0.20 (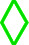), and 0.10 (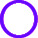) at *C* = 162 mM. The solid lines show the best fitting curves of *k*_r_ with eqn (7) as used in (f) according to eqn (5). (d) The *σ*_e_-dependent *P*_rup_ (60 s) values for *C* = 62 mM (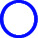), *C* = 162 mM (□), and *C* = 262 mM (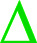) using *X* = 0.40. The solid lines show the best fitting curves of *k*_r_ with eqn (7) as used in (g) according to eqn (5). (e) The time-dependent fraction, *P*_intact_ (*t*), of intact 10%DOPG/90%DOPC-GUVs at *σ*_e_ = 6.5, 7.0 and 7.5 mN m^−1^. The solid lines show the best fitting curves using eqn (4). (f) The *σ*_e_-dependent *k*_r_ values for *X* = 0.60 (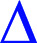), 0.40 (□), 0.20 (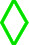), and 0.10 (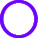) at *C* = 162 mM. The solid lines show the best fitting curves of eqn (5) with *A*_F_ = 5.5 × 10^7^ m^2^ s^−1^ J^−1^, *ω* = 0.49, *B* = 2.27 mN m^−1^, and *Γ* = 13.1 pN for *X* = 0.60 (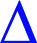); *A*_F_ = 8.8 × 10^6^ m^2^ s^−1^ J^−1^, *ω* = 0.49, *B* = 1.76 mN m^−1^, and *Γ* = 12.1 pN for *X* = 0.40 (□); *A*_F_ = 2.9 × 10^6^ m^2^ s^−1^ J^−1^, *ω* = 0.49, *B* = 0.65 mN m^−1^, and *Γ* = 11.2 pN for *X* = 0.20 (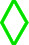); and *A*_F_ = 1.9 × 10^5^ m^2^ s^−1^ J^−1^, *ω* = 0.49, *B* = 0.18 mN m^−1^, and *Γ* = 10.1 pN for *X* = 0.10 (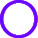). (g) The *σ*_e_-dependent *k*_r_ values for *C* = 62 mM (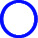), *C* = 162 mM (□), and *C* = 262 mM (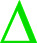) using *X* = 0.40. The solid lines show the best fitting curves of eqn (5) with *A*_F_ = 2.1 × 10^6^ m^2^ s^−1^ J^−1^, *ω* = 0.49, *B* = 4.49 mN m^−1^, and *Γ* = 13.0 pN for *C* = 62 mM (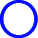); *A*_F_ = 8.8 × 10^6^ m^2^ s^−1^ J^−1^, *ω* = 0.49, *B* = 1.76 mN m^−1^, and *Γ* = 12.1 pN for *C* = 162 mM (□); and *A*_F_ = 8.4 × 10^5^ m^2^ s^−1^ J^−1^, *ω* = 0.49, *B* = 0.48 mN m^−1^, and *Γ* = 10.8 pN for *C* = 262 mM (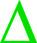). The average values with standard deviations in (c, d, f, and g) were obtained using three independent experiments, each of which was conducted using 18–24 GUVs for each *σ*_e_ value. The images in (c, d, f, and g) have been adapted from ref. 36 with permission from Elsevier B. V., copyright 2020.

In this section, we describe the results of the constant-electric-tension (*σ*_e_)-induced rupture of GUVs with different surface charges and salt concentrations in buffer. The inside of the GUVs was buffer containing 0.10 M sucrose and the outside of the GUVs was buffer containing 0.10 M glucose. The surface charge density of the membranes of the GUVs was varied by changing the DOPG lipid mole fraction (*X*) at a salt concentration *C* of 162 mM in buffer. 40%DOPG/60%DOPC-GUVs (here % indicates mol%, *X* = 0.40) were prepared by varying the *C* in the buffer. The technique to apply the constant electric tension is described in Section 2.2. First, a constant electric tension *σ*_e_ of ∼0.5 mN m^−1^ was applied to a single ‘10%DOPG/90%DOPC-GUV’ (*X* = 0.10) for a few seconds (∼10 s), and then the tension was increased to a specific value, *i.e.*, *σ*_e_ = 6.5 mN m^−1^. Under the applied tension, the GUV remained intact until 37.8 s and then ruptured (Fig. 6(a)i). The rupture occurred due to the formation of nanopores in the membrane, whose radius increased very rapidly.^8,55^ A second GUV was investigated under the same conditions (Fig. 6(a)ii), and rupture occurred after 15.6 s. When the same experiment was applied to several ‘single GUVs’ (number of examined GUVs, *n* = 18–24) at the same tension, rupture occurred at different times, indicating stochastic rupture of the GUVs (Fig. 6(b)). Fig. 6(c) shows the *σ*_e_-dependent probability of rupture before a time of 60 s, *P*_rup_ (60 s) for *X* = 0.10 at a salt concentration *C* of 162 mM, which increased with the tension. The rupture of GUVs was also investigated for various *X* values with a fixed salt concentration (Fig. 6(c)), and in all cases, *P*_rup_ (60 s) increased with *σ*_e_. However, as the value of *X* was increased from 0.10 to 0.60, the tension required to obtain a given value of *P*_rup_ (60 s) became smaller. Fig. 6(d) shows the *σ*_e_-dependent *P*_rup_ (60 s) for different salt concentrations with *X* = 0.40; a lower tension was required to achieve the same *P*_rup_ (60 s) as the salt concentration was decreased. Thus, with increasing electrostatic effects (increased surface charge density or decreased salt concentration), the probability of rupture increased.^36^

To calculate the rate constant (*k*_r_) of rupture, the time-dependent fraction of intact GUVs (*P*_intact_ (*t*)) out of all the examined GUVs was used. Fig. 6(e) shows the time course of *P*_intact_ (*t*) for 10%DOPG/90%DOPC-GUVs at *σ*_e_ = 6.5 mN m^−1^, which was well fitted with eqn (4) (Fig. 6(e)). From the fitted curve, the value of *k*_r_ was obtained as 9.9 × 10^−3^ s^−1^. Similar experiments were performed at *σ*_e_ = 7.0 and 7.5 mN m^−1^, and a faster decrease in *P*_intact_ (*t*) was observed with increasing *σ*_e_. The corresponding values of *k*_r_ were 1.9 × 10^−2^ s^−1^ and 0.8 × 10^−1^ s^−1^. The electric-tension (*σ*_e_)-dependent rate constant of rupture for *X* = 0.10 is presented in Fig. 6(f). Similar experiments were carried out for various *X* values at *C* = 162 mM and for various *C* values at *X* = 0.40. The *σ*_e_-dependent *k*_r_ for various *X* values is shown in Fig. 6(f) and that for various *C* values is shown in Fig. 6(g), in which *k*_r_ increased with *σ*_e_. As a lower tension was required to obtain a given rate constant, the mechanical stability of the membranes greatly decreased with higher anionic lipid content in the membranes or lower salt concentration in the buffer.

The rate constant (*k*_r_) of rupture of the GUVs was determined theoretically using the mean first passage time (MFPT) approach^77–79^ as follows:^37,39^5
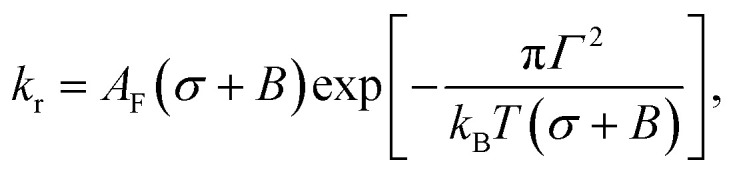
where 
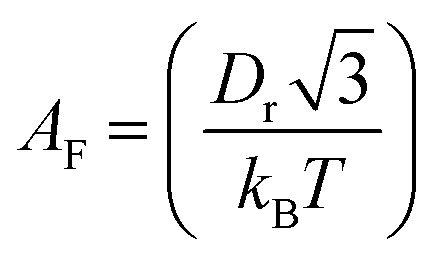
 is the pre-exponential factor, *D*_r_ is the diffusion coefficient of a particle in *r*-phase space, *k*_B_ is the Boltzmann constant and *T* is the absolute temperature. The fitting parameters of eqn (5) are *A*_F_ and line tension (*Γ*). The electrostatic term *B* is defined as follows:^36,80^6
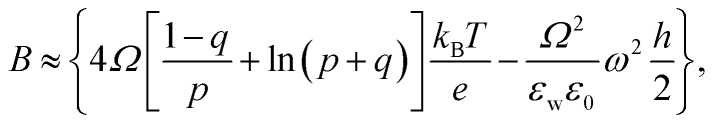
where *h* is the height of the prepore (= 4 nm), the surface charge density *Ω* = *eX*/*A*, *e* is the electronic charge, *X* is the anionic lipid fraction, *A* is the cross-sectional area of the anionic lipid (=72.5 Å^2^ per molecule), *ε*_w_ is the relative dielectric constant of water, *ε*_0_ is the permittivity of free space, *p* = 2π*λ*_B_*X*/*κA* and 
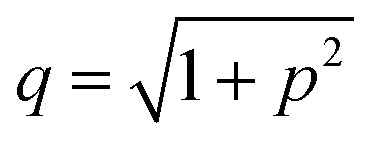
, 
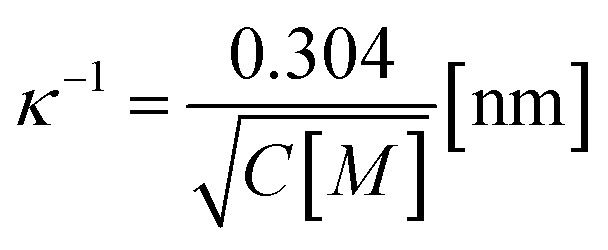
 is the Debye length, *λ*_B_ is the Bjerrum length in water, *λ*_B_ = *e*^2^/4π*k*_B_*Tε*_0_*ε*_*w*_ = 0.716 nm at 25 °C, and *ω* is the fitting parameter, which is the ratio of the surface charge density on the prepore wall to that of the GUV surface. The probability of rupture is defined as follows:

7 *P*_rup_(*σ*,*t*) = 1 − exp(−*k*_r_*t*)

The experimental data (Fig. 6(f)) were fitted using eqn (5), and line tension (*Γ*) values 13.1, 12.1, 11.2 and 10.1 pN were obtained for *X* = 0.60, 0.40, 0.20 and 0.10, respectively, at a fixed *C* of 162 mM. Similarly, line tension values of 13.0, 12.1 and 10.8 pN were obtained for *C* = 62, 162 and 262 mM, respectively, for a fixed *X* value of 0.40 (Fig. 6(g)). These analyses clearly show that the line tension increased with the electrostatic effects (increased surface charge density or decreased salt concentration). The values of *Γ* for various membrane compositions with different bathing solutions are provided in Table 1.

**Table 1:** Line tension values of various membrane compositions under different conditions

Membrane composition (% indicates mol%)	Medium	Measuring technique	Line tension *Γ* (pN)
DOPC	100 mM sucrose inside, 100 mM glucose outside	Micropipette	10.5 (ref. 39)
	200 mM sucrose inside, 200 mM glucose outside		11.5 (ref. 5)
	100 mM sucrose inside, 100 mM glucose outside		11.6 ± 0.2 (ref. 55)
	100 mM sucrose inside, 100 mM glucose outside	Pore closure dynamics and light-induced poration	6.9 ± 0.42 (ref. 48) [Sigma-Aldrich lipid], 20.7 ± 3.5 (ref. 48) [Avanti polar lipid]
	100 mM sucrose inside, 100 mM glucose outside		13–18 (ref. 49)
	240 mM sucrose inside, 260 mM glucose and 1 mM NaCl outside	Pore closure dynamics and electroporation	27.7 ± 2.5 (ref. 7)
	KCl and CaCl_2_ solutions	Electroporation of black lipid membranes	25 (ref. 67)
EggPC	140 mM NaCl, 20 mM HEPES, 1 mM EDTA, pH 7	Pore closure dynamics and electroporation	14.2 ± 0.7 (ref. 83)
diC13:0 PC (DTPC)	200 mM sucrose inside, 200 mM glucose outside	Micropipette	3.1 (ref. 38)
C18:0/1 PC (SOPC)	200 mM sucrose inside, 200 mM glucose outside	Micropipette	8.6 (ref. 38)
SOPC	547 mOsM sucrose inside, 558 mOsM glucose solution with 0.1% albumin outside	Micropipette and electroporation	9.2 ± 0.7 (ref. 75)
diC8:2 PC	200 mM sucrose inside, 200 mM glucose outside	Micropipette	3.25 (ref. 38)
diC22:1 PC	200 mM sucrose inside, 200 mM glucose outside	Micropipette	15 (ref. 38)
10%DOPG/90%DOPC	10 mM PIPES, pH 7.0, 1 mM EGTA, 150 mM NaCl (total salt: 162 mM)	Electroporation	10.1 ± 0.1 (ref. 36)
10%DOPG/90%DOPC	10 mM PIPES, pH 7.0, 1 mM EGTA, 150 mM NaCl (total salt: 162 mM)	Micropipette	10.4 (ref. 80)
20%DOPG/80%DOPC	10 mM PIPES, pH 7.0, 1 mM EGTA, 150 mM NaCl (total salt: 162 mM)	Electroporation	11.2 ± 0.3 (ref. 36)
40%DOPG/60%DOPC	10 mM PIPES, pH 7.0, 1 mM EGTA, 150 mM NaCl (total salt: 162 mM)	Electroporation	12.1 ± 0.1 (ref. 36)
40%DOPG/60%DOPC	10 mM PIPES, pH 7.0, 1 mM EGTA, 150 mM NaCl (total salt: 162 mM)	Micropipette	11.4,^80^ 12.4 ± 0.2 (ref. 55)
60%DOPG/40%DOPC	10 mM PIPES, pH 7.0, 1 mM EGTA, 150 mM NaCl (total salt: 162 mM)	Electroporation	13.1 ± 0.4 (ref. 36)
40%DOPG/60%DOPC	10 mM PIPES, pH 7.0, 1 mM EGTA, 50 mM NaCl (total salt: 62 mM)	Electroporation	13.0 ± 0.2 (ref. 36)
40%DOPG/60%DOPC	10 mM PIPES, pH 7.0, 1 mM EGTA, 250 mM NaCl (total salt: 262 mM)	Electroporation	10.8 ± 0.3 (ref. 36)
40%DOPG/60%DOPC	10 mM PIPES, pH 7.0, 1 mM EGTA, 300 mM NaCl (total salt: 312 mM)	Micropipette	10.5 (ref. 80)
46%DOPG/39%DOPC/15%chol	10 mM PIPES, pH 7.0, 1 mM EGTA, 150 mM NaCl (total salt: 162 mM)	Electroporation	12.9 (ref. 84)
43%DOPG/28%DOPC/29%chol	10 mM PIPES, pH 7.0, 1 mM EGTA, 150 mM NaCl (total salt: 162 mM)	Electroporation	13.8 (ref. 84)
40%DOPG/20%DOPC/40%chol	10 mM PIPES, pH 7.0, 1 mM EGTA, 150 mM NaCl (total salt: 162 mM)	Electroporation	14.6 (ref. 84)
DOPC/chol (5 : 1 mol)	240 mM sucrose inside, 260 mM glucose and 1 mM NaCl outside	Pore closure dynamics and electroporation	36.4 ± 1.9 (ref. 7)
50%SOPC/50%chol	547 mOsM sucrose inside, 558 mOsM glucose solution with 0.1% albumin outside	Micropipette and electroporation	30.5 ± 1.2 (ref. 75)
50%SOPC/50%chol	200 mM sucrose inside, 200 mM glucose outside	Micropipette	20.5 (ref. 38)
50%SM/50%chol	200 mM sucrose inside, 200 mM glucose outside	Micropipette	23 (ref. 38)
DOPC/DOPE (5 : 1 mol)	240 mM sucrose inside, 260 mM glucose and 1 mM NaCl outside	Pore closure dynamics and electroporation	15.6 ± 1.3 (ref. 7)

### 3.2. Effects of surface charge density and salt concentration on the constant-mechanical-tension-induced rupture of GUVs

**Fig. 7:**
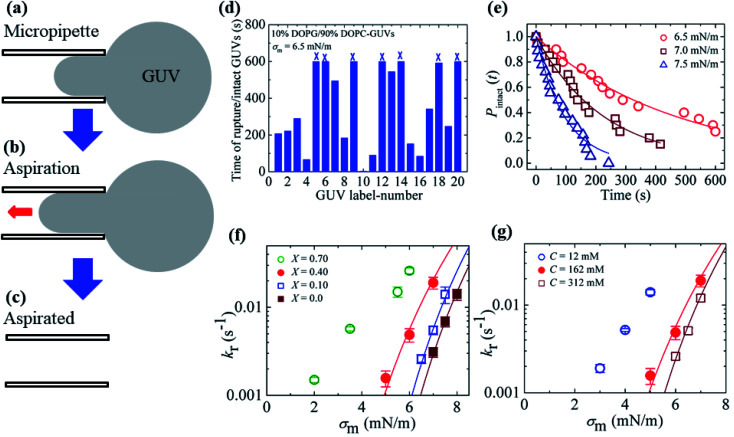
The constant-mechanical-tension (*σ*_m_)-induced rupture of GUVs under various conditions. (a) A ‘single GUV’ aspirated with a micropipette using ∼0.5 mN m^−1^. (b) Significant aspiration due to high membrane tension. (c) The complete aspiration of the GUV into the micropipette. (d) The time of stochastic rupture and intact status of several ‘single 10%DOPG/90%DOPC-GUVs’ (*n* = 20) at *σ*_m_ = 6.5 mN m^−1^. The crosses (×) above the bars indicate that the GUV was intact at 600 s. (e) The time-dependent fraction, *P*_intact_ (*t*), of intact 10%DOPG/90%DOPC-GUVs at *σ*_m_ = 6.5, 7.0, and 7.5 mN m^−1^. The solid lines show the best fitting curves based on eqn (4). (f) The *σ*_m_-dependent *k*_r_ values for *X* = 0.70 (○), 0.40 (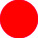), 0.10 (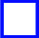), and 0.0 (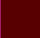) at *C* = 162 mM. The solid lines show the best fitting curves of eqn (5) with *D*_r_ = 165 nm^2^ s^−1^ and *ω* = 0.45. The best fitting curves are shown for *X* = 0.40 (*Γ* = 11.4 pN), *X* = 0.10 (*Γ* = 10.4 pN), and *X* = 0.0 (*Γ* = 10.5 pN). (g) The *σ*_m_-dependent *k*_r_ values for *C* = 12 mM (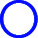), *C* = 162 mM (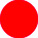), and *C* = 312 mM (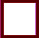) using *X* = 0.40. The solid lines show the best fitting curves of eqn (5) with *D*_r_ = 165 nm^2^ s^−1^ and *ω* = 0.45. The best fitting curves are shown for *C* = 162 mM (*Γ* = 11.4 pN), and 312 mM (*Γ* = 10.5 pN). The average values with standard deviations shown in f and g were determined using three independent experiments, each of which was conducted using 18–24 GUVs for each *σ*_m_ value. The images in f and g have been adapted from ref. 80 with permission from American Physical Society, copyright 2015.

In Section 3.1, constant electric tension (*i.e.*, the electroporation technique) was used to induce the rupture of GUVs. In this section, a similar experiment was performed using mechanical tension (*i.e.*, the micropipette aspiration technique). The technique of constant mechanical tension (*σ*_m_) is described in Section 2.3. First, a single ‘10%DOPG/90%DOPC-GUV’ (anionic lipid mole fraction *X* = 0.10) was held at the tip of a micropipette for 2 min using slight aspiration (equivalent tension *σ*_m_ of ∼0.5 mN m^−1^) (Fig. 7(a)); the GUV was then quickly (∼10 s) aspirated to a specific level of tension (*i.e.*, *σ*_m_ = 6.5 mN m^−1^) and held at this tension for 10 min (Fig. 7(b)). After some time, the GUV was aspirated suddenly into the micropipette (Fig. 7(c)). The rupture occurred due to the formation of a nanopore in the membrane, whose radius increased rapidly. The same experiment was repeated for many single ‘10%DOPG/90%DOPC-GUVs’ at the same tension, and rupture occurred at different times, indicating stochastic rupture (Fig. 7(d)). The time-dependent fraction of intact GUVs, *P*_intact_ (*t*), out of all the studied GUVs at *σ*_m_ = 6.5 mN m^−1^ is shown in Fig. 7(e), and was well fitted by eqn (4), and hence, the rate constant of rupture was found to be *k*_r_ = 2.1 × 10^−3^ s^−1^. Similar experiments were performed at *σ*_m_ = 7.0 and 7.5 mN m^−1^, which resulted in a faster decrease in *P*_intact_ (*t*) with increasing *σ*_m_. Thus, corresponding *k*_r_ values of 4.4 × 10^−3^ s^−1^ and 1.1 × 10^−2^ s^−1^ were obtained. The same steps were followed to determine the *σ*_m_-dependent *k*_r_ values for several *X* values at *C* = 162 mM (Fig. 7(f)), and for several *C* values at *X* = 0.40 (Fig. 7(g)). For a particular tension, the rate constant increased with increasing anionic lipid content as well as with decreasing salt concentration in the buffer. These results suggested that the mechanical stability of the membrane greatly decreased with electrostatic effects (increased surface charge density or decreased salt concentration). The experimental *σ*_m_*vs. k*_r_ result was fitted with eqn (5) and line tension values of *Γ* = 11.4, 10.4 and 10.5 pN for *X* = 0.40, 0.10 and 0.0, respectively, were obtained for a salt concentration *C* of 162 mM. Similarly, line tensions of 11.4 and 10.5 pN were obtained for *C* = 162 and 312 mM, respectively, for an anionic lipid fraction *X* of 0.40. The line tension increased with increasing *X* or with decreasing *C*. The line tension values obtained using the micropipette technique were very similar to those obtained using the electroporation technique. The line tensions for various membrane compositions under different conditions are shown in Table 1.

If the anionic lipid content in the membranes is increased, the repulsive force between the lipid molecules will increase and hence increase the electrostatic effects. Similarly, if the salt concentration in the buffer increases, the ions in solution will shield the surface of the lipid membranes to a greater extent, and hence lessen the electrostatic effects.^81,82^ These explanations support the results described in Sections 3.1 and 3.2. As the electrostatic effects increased (*i.e.*, increased anionic lipid content or decreased ionic salt content), the probability of rupture increased, along with the rate constant of rupture. Therefore, the results demonstrated in Fig. 6 and 7 clearly show that the anionic lipid content and salt concentration play an important role in the rupture processes of GUVs under constant tension.

### 3.3. Energy profile for the rupture of GUVs

**Fig. 8:**
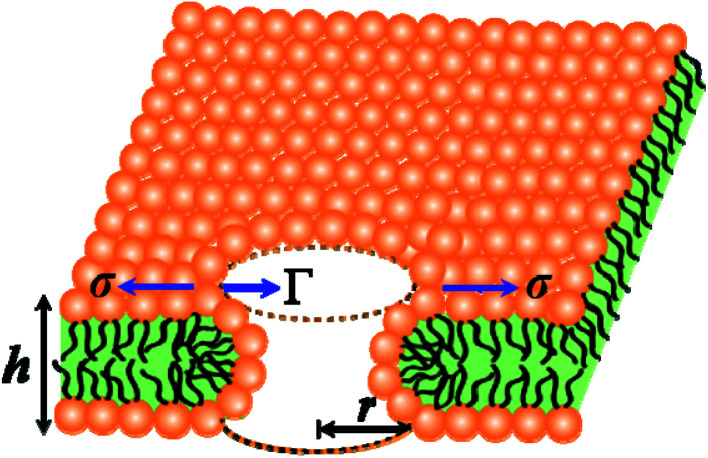
An illustration of the toroidal structure of a prepore with radius *r*.

In the liquid crystalline phase or liquid disordered phase, the lipid bilayer fluidity (which is associated with the diffusion coefficient of lipid molecules) and the molecular movement of lipids are large. Due to these properties, rarefaction in several regions (*i.e.*, lower lipid density) and condensation (*i.e.*, higher lipid density) occur transiently, as the lipid bilayer fluctuation is large, which is driven by thermal energy. Such rarefactions are treated as prepores with hydrophobic and hydrophilic structures.^44,85–91^ Prepores are essentially narrow channels through which the internal contents of the vesicle, such as fluorescent probes, cannot pass significantly into the outer environment; the lifetime of prepores is very short. Once the prepores are converted to pores, such probes pass through the water channel in the lipid bilayer. The orientational change in the of lipids is insignificant in hydrophobic prepores; the walls of the prepores are composed of the hydrocarbon chains of the lipids and are in contact with water inside the prepores. In this situation, the line tension of the prepores is primarily due to the hydrophobic interaction between the lipid chains and water. Hydrophilic prepores are widely considered to have a toroidal structure in which the outer and inner monolayer of the bilayer bend and connect to each other at the prepores in a toroidal fashion.^48,85,87,89^ The orientation of the lipid molecules changes in such a way that the inner wall of the prepores composed of lipid heads is contact in with the water channel (Fig. 8). The bending energy of the monolayer determines the line tension of hydrophilic prepores.^92^

In hydrophilic prepores, part of the lipid chains in the walls of the prepores are in contact with water (*i.e.*, hydrophobic interaction) due to the large deformation in the lipid structure. This hydrophobic interaction increases the line tension. According to the observations of Glaser *et al.*,^85^ when the radius of a prepore is very small, it is hydrophobic, and with increasing size, it becomes hydrophilic. However, in a molecular dynamics (MD) simulation, hydrophobic prepores were not observed.^87,89^ Hence, one can consider only the hydrophilic prepores. Based on these discussions, when a prepore is formed in a lipid membrane in the presence of external tension (here *σ* = *σ*_e_ = *σ*_m_), the total free energy of the system changes by an additional free energy (*i.e.*, prepore free energy) and is defined as:^93,94^*U*(*r*,*σ*) = 2π*rΓ* − π*r*^2^*σ*, where the term 2π*rΓ* is due to the line tension (*Γ*) of the prepore edge and −π*r*^2^*σ* is due to the lateral tension (*σ*) in the membrane. GUV rupture occurs as the pore radius becomes very large within a very short time. If the radii of the prepores reach the critical value at different times, stochastic rupture occurs, the analysis of which gives the rate constant of GUV rupture. The prepore free energy for charged membranes is expressed as follows:^80^

8 *U*(*r*,*σ*) = 2π*Γr* − π*r*^2^(*σ* + *B*)

At the critical radius of the prepore, 
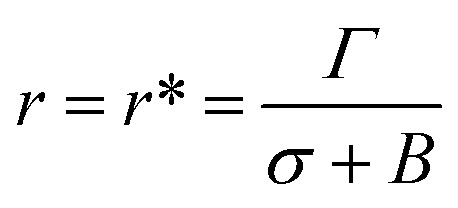
, the energy barrier of the prepore free energy is expressed as follows:9
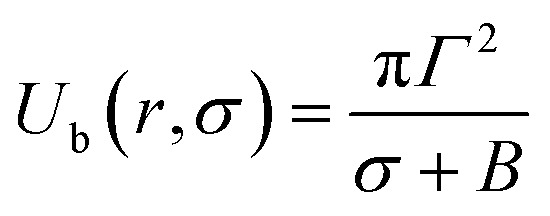


**Fig. 9:**
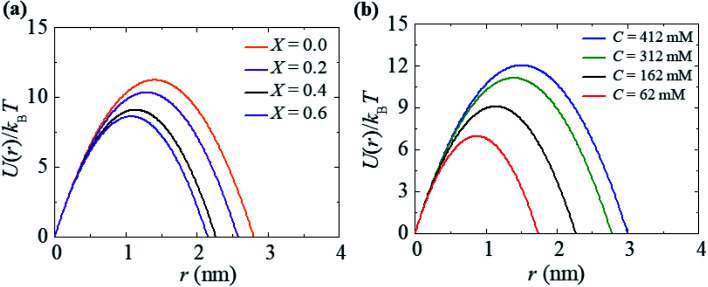
The prepore free energy profiles for different values of (a) *X* with *C* = 162 mM and (b) *C* with *X* = 0.40 at *σ* = 7.5 mN m^−1^. *U*(*r*) was calculated according to eqn (8) using *Γ* = 10.5 pN and *ω* = 0.49.

For a neutral membrane, *B* = 0. According to eqn (9), the main factor of rupture depends on the energy barrier *U*_b_ (*σ*, *B*, *Γ*). For a fixed line tension, *U*_b_(*r*, *σ*, *B*) decreases as *X* increases, and consequently, *k*_r_ increases (Fig. 9(a)). Similarly, *U*_b_(*r*, *σ*, *B*) decreases as *C* decreases, and therefore, *k*_r_ increases (Fig. 9(b)). Hence, the prepore free energy profile (Fig. 9) demonstrates that the theory of a tension-dependent rate constant of rupture reasonably explains the experimental results. Due to the presence of 10–20% anionic lipids in plasma membranes,^95^ the electrostatic interactions have important implications for the rupture of vesicles induced by electric or mechanical tension. Additionally, since the mechanical stability of the membrane greatly depends on the surface charge and the bathing salt, the electrostatic interactions must be taken into consideration for any realistic scenario, such as cell ablation using irreversible electroporation.

A theoretical model of pore formation in lipid membranes under electrical and mechanical tension describes the pore formation trajectory.^44^ In this model, the optimal shape of the pore surface is formed by minimization of the energy in each state of pore creation and pore growth. This model proposes that the metastability of the hydrophilic pores occurs due to compensation of the effects of positive curvature and negative curvature at the pore edge, and was investigated using MD simulations.^96^

As mentioned above, when the radius of a prepore crosses the critical value and subsequently overcomes the energy barrier, the prepore is converted into a transmembrane pore. In the presence of constant electric or mechanical tension, the trend for all ‘single GUVs’ to overcome the energy barrier is stochastic in nature. For this reason, the rupture of several ‘single GUVs’ is stochastic under constant tension, which means that the pore formation occurs at different times even though the tension is the same.

The pore dynamics, such as the opening and closing of transient pores, as well as the bursting of pores, have been investigated previously.^4^ Constant tension was induced by adhering the GUVs to a glass substrate and by optical illumination. The bursting mechanism of the GUVs due to the strongly attractive glass surface was irreversible. Using optical illumination, a transient pore was formed in the membranes, which then closed with time. The size of the pores varied depending on the viscosity of the liquid. In the case of higher viscosity liquids, the leakage of the internal contents of the GUVs was slower, resulting in a larger pore. In contrast, when the viscosity was lower, the leakage was faster, and hence produced a smaller pore. Later, the line tension was measured by investigating the closure dynamics of a single pore induced by optical illumination.^48^ The authors also studied the cascades of transient pores in GUVs. As the illumination time was extended, a transient pore first opened and then resealed within a few seconds by ejecting internal fluid, resulting in a decrease in the GUV radius. The illumination was continued, and after an interval, another pore opened and closed in the same manner. The subsequent pores followed the mechanism of the previous ones. The interval between the successive pores (*i.e.*, induction time) increased and the vesicle radius decreased with increasing illumination time. Additionally, the oscillatory phase separation behavior of the membranes prepared by lipid mixtures when the GUVs were subjected to an osmotic stress was investigated.^97^ A series of swell–burst cycles occurred due to the osmotic gradient across the membrane. Domains of liquid ordered phase and liquid disordered phase were formed in the membranes in the swollen state, which were then eliminated (*i.e.*, the membrane became uniform) in the shrunken state. The shrinkage occurred due to the release of membrane tension by ejecting internal fluid through the pores. This type of oscillation (swell–burst cycles) continued until the osmotic gradient became zero. Moreover, various mechanisms of glass-supported bilayer formation *via* the rupture of a ‘single GUV’ due to the lipid–glass adhesion (*i.e.*, tension due to adhesion) were investigated.^98^ In the presence of a high concentration of Texas Red DHPE (TR-DHPE) in the membranes of GUVs, the rupture mechanism occurred in two different ways. One involved asymmetric patches (almost heart-shaped) in which the pore initiation point was situated near the adsorbed region, and another involved symmetric patches (circular) in which the pore initiation point was situated near the apex of the adsorbed structure. In contrast, the asymmetric pathway was followed for low concentrations of TR-DHPE and in the absence of TR-DHPE. Later, the rupture dynamics of GUVs caused by interaction with hydrophobic and hydrophilic surfaces on a glass substrate were investigated.^99^ For the hydrophobic surface, a pore was first initiated and then closed by expelling internal fluid, which resulted in a smaller vesicle than the original one. The lipid molecules of the inner and outer monolayer exchanged positions in the smaller vesicle, leaving a lipid monolayer on the hydrophobic surface. In contrast, for a hydrophilic surface, after the pore resealed, a flat bilayer was formed on the glass surface without the exchange of lipid molecules between the inner and outer monolayer of the GUV with a smaller diameter than the interacting one. Furthermore, cell growth dynamics (cultured HeLa cells) were investigated by measuring the ‘dry mass’ in presence of different concentrations of glucose in the medium.^100^ Higher concentrations of glucose led to a higher percentage of cell growth in the culture medium.

### 3.4. Activation energy of the constant-mechanical-tension-induced rupture of GUVs

**Fig. 10:**
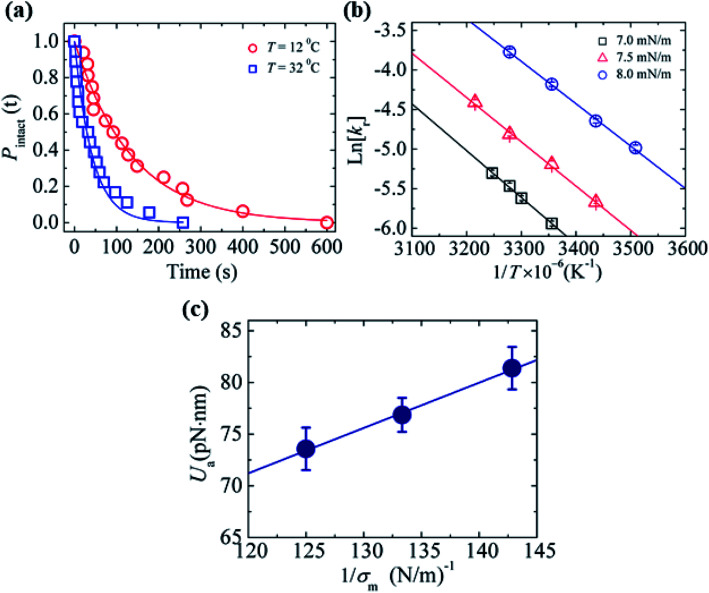
The effects of temperature on the constant-mechanical-tension (*σ*_m_)-induced rupture of DOPC-GUVs. (a) The time-dependent *P*_intact_ (*t*) values at temperatures of *T* = 12 °C (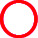) and *T* = 32 °C (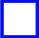) at *σ*_m_ = 8.0 mN m^−1^. The solid lines represent the best fit curves based on eqn (4). (b) The relationship between ln*k*_r_ and 1/*T* at *σ*_m_ = 7.0 mN m^−1^ (□), 7.5 mN m^−1^ (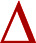), and 8.0 mN m^−1^ (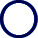). Average values of *k*_r_ with standard error for each temperature were determined using three independent experiments, each of which was performed using 20 GUVs for each tension. The solid lines show the best linear fits. (c) The relationship between *U*_a_ and 1/*σ*_m_. The error bars show standard error. The solid line indicates the best fit curve using eqn (10). The images in a–c have been adapted from ref. 101 with permission from AIP Publishing LLC, copyright 2015.

In Section 3.3, it was observed that the energy barrier is one of the important factors in the rupture of GUVs under electric and mechanical tension. In this section, we describe the results of the measurement of the activation energy (*U*_a_) of rupture of DOPC-GUVs. *U*_a_ is the minimum energy required for the rupture of GUVs, which is a similar concept to the energy barrier (*U*_b_). The rate constant for any reaction can be expressed as:^101^*k*_r_ = *A*_c_exp(−*U*_a_/*k*_B_*T*), where *A*_c_ is the prefactor, *k*_B_ is the Boltzmann constant and *T* is the absolute temperature. First, the rate constant (*k*_r_) of constant-mechanical-tension (*σ*_m_)-induced rupture of GUVs was calculated using the same method as described in Section 3.2. Then, the activation energy was calculated using the above equation. Fig. 10(a) shows the time course of the fraction of intact GUVs, *P*_intact_ (*t*), in the presence of a *σ*_m_ of 8.0 mN m^−1^ at temperatures *T* of 12 and 32 °C. The solid line (Fig. 10(a)) shows the best fitting theoretical curve using eqn (4), from which the rate constant of rupture was determined. The values obtained were *k*_r_ = 7.3 × 10^−3^ s^−1^ at 12 °C and 2.4 × 10^−2^ s^−1^ at 32 °C, which shows that *k*_r_ increased with temperature. Fig. 10(b) shows the graph of ln*k*_r_*versus* 1/*T*. The activation energies were obtained from the slope of the linear fit, and were *U*_a_ = 73.7 ± 1.9 pN nm (= 17.9 ± 0.5 *k*_B_*T*) for *σ*_m_ = 8.0 mN m^−1^, *U*_a_ = 81.5 ± 1.9 pN nm (= 19.8 ± 0.5 *k*_B_*T*) for *σ*_m_ = 7.0 mN m^−1^ and *U*_a_ = 77.1 ± 1.4 pN nm (= 18.8 ± 0.4 *k*_B_*T*) for *σ*_m_ = 7.5 mN m^−1^. The dependence of the activation energy (*U*_a_) on 1/*σ*_m_ is shown in Fig. 10(c), which was fitted linearly using the following equation:10
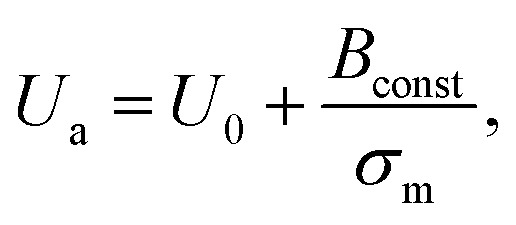
where *U*_0_ and *B*_const_ are constants that do not depend on the tension. From the intercept of the line, a value of *U*_0_ = 19 ± 3 pN nm (= 4.7 ± 0.6 *k*_B_T) was obtained for the DOPC-GUVs. Here, *U*_0_ was considered to be the nucleation free energy to form a hydrophilic prepore from a hydrophobic prepore. By comparing eqn (10) and (9) (*B* = 0 for DOPC-GUVs), the value of *Γ* was calculated to be 11.6 ± 0.2 pN for the DOPC-GUVs.

A similar investigation was performed using 40%DOPG/60%DOPC-GUVs, and values of *U*_0_ = 9.0 ± 0.4 pN nm and *Γ* = 12.4 ± 0.2 pN were obtained.^55^ The line tension for the 40%DOPG/60%DOPC-GUVs was slightly higher than that of the DOPC-GUVs, which was in agreement with the literature.^80^ Therefore, these investigations provided important information to explain the mechanism of the constant-mechanical-tension-induced rupture of GUVs. However, more investigations are needed to understand the complete mechanism.

### 3.5. Effects of the hydrocarbon chain length of lipids on the constant-mechanical-tension-induced rupture of GUVs

The effect of the hydrocarbon chain length of the lipids on the mechanical properties of the lipid membranes was investigated using constant-mechanical-tension (*σ*_m_)-induced rupture of GUVs. In this case, 10%DLPG/90%DTPC-GUVs (% indicates mol%) and 40%DLPG/60%DTPC-GUVs were prepared in buffer (see Section 3.1). The rate constant (*k*_r_) of the constant-mechanical-tension (*σ*_m_)-induced rupture of the DLPG/DTPC-GUVs was compared with the corresponding rate constant for the DOPG/DOPC-GUVs. The carbon chain of DLPG is 12 : 0 and that of DTPC is 13 : 0. In contrast, the carbon chain for both DOPG and DOPC is 18 : 1. The peak-to-peak headgroup thickness of DTPC is 3.41 ± 0.05 nm and that of DOPC is 3.69 ± 0.04 nm.^102^ The rate constant of rupture was determined at various tensions for the DLPG/DTPC-GUVs as described in Section 3.2.

**Fig. 11:**
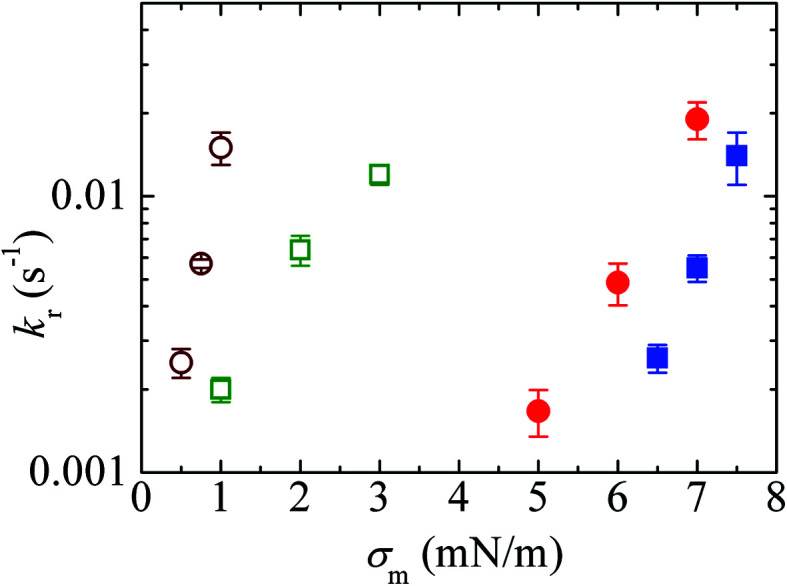
The effects of the constant-mechanical-tension (*σ*_m_)-induced rupture of GUVs. The *σ*_m_-dependent *k*_r_ values for 40%DLPG/60%DTPC-GUVs (○), 10%DLPG/90%DTPC-GUVs (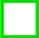), 40%DOPG/60%DOPC-GUVs (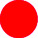), and 10%DOPG/90%DOPC-GUVs (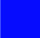). Mean *k*_r_ values with standard errors for each tension were determined using three independent experiments, each of which was conducted using 20 GUVs for each tension. This figure has been adapted from ref. 103 with permission from American Chemical Society, copyright 2016.

Fig. 11 shows the *σ*_m_-dependent *k*_r_ values for the 10%DLPG/90%DTPC-GUVs and 40%DLPG/60%DTPC-GUVs. The *k*_r_ values for both GUVs increased with *σ*_m_. In addition, for a similar rate constant, the tension required to rupture the 40%DLPG/60%DTPC-GUVs was smaller than that required for the 10%DLPG/90%DTPC-GUVs (Fig. 11). This result was reasonably explained by the electrostatic interaction effect.^80,104,105^ For comparison, the *k*_r_ data for the 10%DOPG/90%DOPC-GUVs and 40%DOPG/60%DOPC-GUVs are also shown in Fig. 11. The tension required to induce rupture in the DLPG/DTPC-GUVs was much smaller than those required for the DOPG/DOPC-GUVs. As an example, for the 40%DLPG/60%DTPC-GUVs, tensions of 0.5, 0.75 and 1.0 mN m^−1^ were applied, whereas for the 40%DOPG/60%DOPG-GUVs tensions of 5.0, 6.0 and 7.0 mN m^−1^ were applied to obtain similar rate constants. These results indicated that the DLPG/DTPC-GUVs were mechanically less stable than the DOPG/DOPC-GUVs.

### 3.6. Effects of cholesterol on the constant-electric-tension-induced rupture of GUVs

**Fig. 12:**
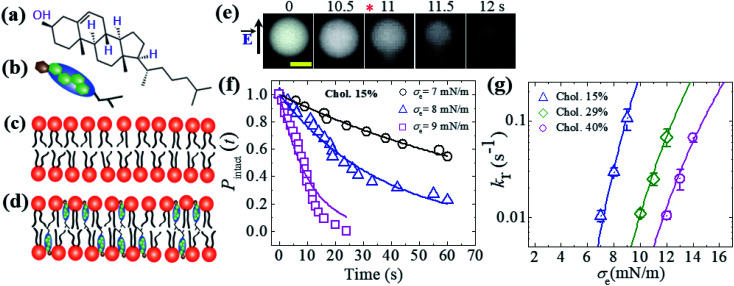
An illustration of cholesterol and the constant-electric-tension (*σ*_e_)-induced rupture of cholesterol-containing GUVs. (a) The structure of cholesterol. Illustrations of (b) cholesterol, (c) a lipid membrane without cholesterol, and (d) a cholesterol-containing lipid membrane. (e) Fluorescence images of the rupture of a ‘single 46%DOPG/39%DOPC/15%chol-GUV’ at *σ*_e_ = 8.0 mN m^−1^. The field direction is shown by the arrow at the left side of the images. The numbers above each image indicate the time in seconds after applying *σ*_e_. The scale bar is 15 μm. The time of rupture is indicated by an asterisk (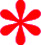). (f) The time-dependent fractions of intact 46%DOPG/39%DOPC/15%chol-GUVs at *σ*_e_ = 7.0, 8.0, and 9.0 mN m^−1^. The solid lines represent the best fitting curves using eqn (4). (g) The constant-electric-tension (*σ*_e_)-dependent *k*_r_ values for 46%DOPG/39%DOPC/15%chol- (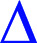), 43%DOPG/28%DOPC/29%chol- (⋄), and 40%DOPG/20%DOPC/40%chol-GUVs (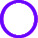). The solid lines show the best fit theoretical curves of eqn (5) with *A*_F_ = 9.27 × 10^5^ m^2^ s^−1^ J^−1^, *ω* = 0.49, *B* = 2.10 mN m^−1^, and *Γ* = 12.9 pN for 15%chol (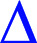); *A*_F_ = 1.47 × 10^5^ m^2^ s^−1^ J^−1^, *ω* = 0.49, *B* = 2.10 mN m^−1^, and *Γ* = 13.8 pN for 29%chol; (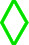) and *A*_F_ = 9.27 × 10^4^ m^2^ s^−1^ J^−1^, *ω* = 0.49, *B* = 2.10 mN m^−1^, and *Γ* = 14.6 pN for 40%chol-content GUVs (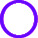). Average values of *k*_r_ with standard deviations for each tension were determined from three independent experiments, each of which was conducted using 18–24 GUVs for each value of *σ*_e_. The images in (e, f, and g) have been adapted from ref. 35 with permission from the European Biophysical Societies' Association, copyright 2020.

So far, we have discussed the constant-electric- and -mechanical-tension-induced rupture of GUVs with various surface charges, salt concentrations and lipid compositions. In this section, the rupture of cholesterol-containing GUV membranes is described. The addition of cholesterol (*i.e.*, chol, *C*_h_) condenses the lipid membranes.^106–109^ The cross-sectional area of anionic lipid is about 72.5 Å^2^ per molecule.^95^ The presence of cholesterol decreases the surface area to 50, 42 and 40 Å^2^ per molecule for 15, 29 and 40 mol% cholesterol, respectively.^110,111^ The cross-sectional area of cholesterol is considered to be approximately half (*i.e.*, 33−38 Å^2^ per molecule) that of lipid molecules.^107^ 46%DOPG/39%DOPC/15%chol- (% indicates mol%), 43%DOPG/28%DOPC/29%chol- and 40%DOPG/20%DOPC/40%chol-GUVs were prepared using the natural swelling method with a surface charge density of approximately −0.16 C m^−2^. The chemical structure of cholesterol and cartoons of cholesterol, a lipid membrane without cholesterol and a cholesterol-containing lipid membrane are illustrated in Fig. 12(a–d). A constant electric tension *σ*_e_ of 8.0 mN m^−1^ was applied to a ‘single 46%DOPG/39%DOPC/15%chol-GUV’ using the method described in Section 2.2, and the GUV was then observed for 60 s (Fig. 12(e)). In this case, the GUVs were observed using a fluorescence microscope; the GUVs encapsulated 1 mM calcein containing 0.10 M sucrose. Under an applied tension, the GUV remained intact with a spherical shape, and rupture occurred at 11 s. The experiment was repeated for several ‘single GUVs’ and rupture occurred stochastically. Fig. 12(f) shows the time course of the fraction of intact GUVs, *P*_intact_ (*t*), at *σ*_e_ = 7.0, 8.0 and 9.0 mN m^−1^, which were well fitted using eqn (4), and the corresponding rate constants (*k*_r_) of rupture were found to be 1.0 × 10^−2^, 2.7 × 10^−2^ and 1.1 × 10^−1^ s^−1^. The *σ*_e_-dependent *k*_r_ values are shown in Fig. 12(g), in which *k*_r_ increased with *σ*_e_. However, higher tension was required to obtain a similar rate constant for higher-cholesterol-content membranes. These results indicated that the addition of cholesterol greatly increased the mechanical stability of the membranes. The experimental data (Fig. 12(g)) was fitted using eqn (5) and line tension values *Γ* of 12.9, 13.8 and 14.6 pN were obtained for the 15%chol, 29%chol and 40%chol GUVs, respectively. Hence, the line tension increased with the cholesterol content. The values of line tension for the different membrane compositions are provided in Table 1.

As the line tension increased with increasing cholesterol content, *U*_b_(*r*, *σ*) increased (from eqn (9)), and consequently, the rate constant of rupture decreased. The prepore energy profile was similar for the oligoarginine-induced poration in the lipid bilayers containing cholesterol.^103^ The constant-current measurements of a planar membrane exhibit constant-intensity current flow through the membranes due to the formation of fluctuating pores. The presence of cholesterol in the lipid bilayer causes an increase in the breakdown potential. The greater stability of cholesterol-containing membranes results from the increased critical pore radius.^112^ MD simulations demonstrated that the pore formation rate was much slower in DOPC membranes containing cholesterol subjected to an electric field due to the substantial increase in membrane cohesion.^113^ The MD simulation also indicated that increasing the cholesterol content from 20 to 50 mol% in lipid:sterol substantially increased the cohesion of the membranes, and hence increased the electroporation threshold.^114^ This increase in the threshold is often linked to the increase in bilayer stiffness.^115^

### 3.7. Effects of constant electric tension on a GUV connected to another GUV

**Fig. 13:**
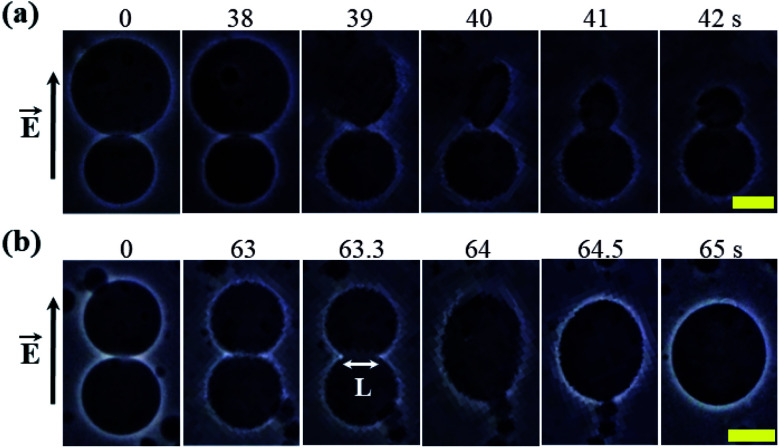
A ‘single 40%DOPG/60%DOPG-GUV’ connected to another GUV of the same membrane composition. (a) The rupture of one GUV while the other remained intact under a constant electric tension *σ*_e_ of 6.2 mN m^−1^. (b) The time course of the electrofusion of GUVs under *σ*_e_ = 4.1 mN m^−1^. The field direction is shown at the left side of each image. The numbers in the images show the time in seconds after applying electric tension. The scale bars in (a and b) are 15 μm. The images in (a and b) have been adapted from ref. 8 with permission from European Biophysical Societies' Association, copyright 2019.

In this section, the effects of constant electric tension (*σ*_e_) on two interconnected 40%DOPG/60%DOPC-GUVs (% indicates mol%) is discussed. With the application of a constant tension *σ*_e_ of 6.2 mN m^−1^, rupture occurred in the bigger GUV at 39 s, but the smaller GUV remained intact (Fig. 13(a)). Following the rupture, a tether-like structure was observed at 41 s, and finally, a smaller vesicle was formed at 42 s (Fig. 13(a)). This type of small vesicle was formed due to the presence of a chelating agent such as EGTA. When a constant electric tension of *σ*_e_ = 4.1 mN m^−1^ was applied to two interconnected similarly sized 40%DOPG/60%DOPC-GUVs, electrofusion occurred (Fig. 13(b)). Simultaneous pore formation at the fusion neck (here, the length of the fusion neck was *L* = 11.9 μm at time = 63.3 s) was responsible for the electrofusion in the GUVs. The two GUVs started to merge into a single GUV at a time of 63.3 s and formed a single GUV at 64 s (Fig. 13(b)). This type of electrofusion was also observed in other investigations.^34,116^

### 3.8. Estimation of membrane tension under different osmotic pressures using constant-electric-tension-induced rupture in GUVs

So far, we have discussed the results of constant-electric- and mechanical-tension-induced rupture of GUVs for different surface charges, salt concentrations, and lipid compositions, as well as that of cholesterol-containing membranes. This section and the following one discuss the estimation of the membrane tension (*σ*_oseq_) at swelling equilibrium under various osmotic pressures (*Π*). The osmolarity of the sucrose inside the 46%DOPG/39%DOPC/15%chol-GUVs (*C*^0^_in_) was 388 mOsm L^−1^. To apply an osmotic pressure, first, 280 μL of a buffer containing 76.6 mM or 72.3 mM glucose was placed in a microchamber. 20 μL of an unpurified 46%DOPG/39%DOPC/15%chol-GUVs suspension containing a 98 mM (= 394 mOsm L^−1^) sucrose solution was transferred into the microchamber; hence, the corresponding glucose concentrations outside the GUVs (*C*_out_) were 78 mM (=373 mOsm L^−1^) and 74 mM (=369 mOsm L^−1^). The corresponding osmolarity differences between the inside and outside of the GUVs were Δ*C*^0^ = *C*^0^_in_ − *C*_out_ = 15 and 19 mOsm L^−1^. Due to the concentration gradient, the GUVs swelled as water molecules from the glucose solution passed into the inside of the GUVs. The osmotic pressure created lateral membrane tension in the GUVs. Similarly, the osmolarity values of the glucose solution were 80 mM (= 375 mOsm L^−1^) and 76 mM (= 371 mOsm L^−1^) for 40%DOPG/60%DOPC-GUVs. The corresponding osmolarity differences were 13 and 17 mOsm L^−1^ for the 40%DOPG/60%DOPC-GUVs.

The theory of membrane tension due to osmotic pressure has been described previously.^9^ A ‘single GUV’ is considered to have an initial radius of *r*_0_, and its initial inside osmolarity is *C*^0^_in_ (mOsm L^−1^). If the GUV is transferred into a hypotonic solution with *C*_out_ (mOsm L^−1^), an osmotic pressure is induced in the GUV, and as a result, the radius of the GUV increases to Δ*r*_eq_ and the corresponding membrane tension is *σ*_oseq_ at swelling equilibrium. The expression for this is *Π* = *RT*Δ*C*^0^, where Δ*C*^0^ is the difference in osmolarity, *R* is the gas constant and *T* is the absolute temperature. The membrane tension at swelling equilibrium is defined as follows:11
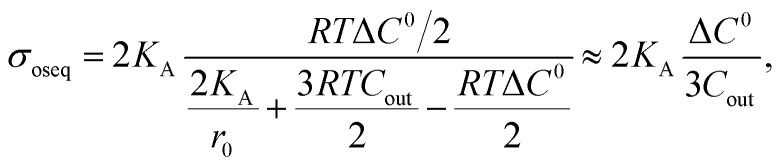
where *K*_A_ is the area compressibility modulus, which was 154 ± 4 mN m^−1^ for the 46%DOPG/39%DOPC/15%chol-GUVs^11^ and 141 ± 5 mN m^−1^ for 40%DOPG/60%DOPC-GUVs.^12^

**Fig. 14:**
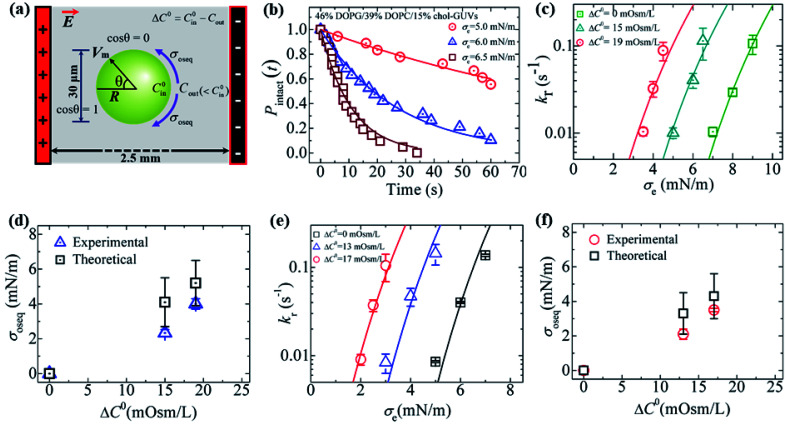
Constant-electric-tension (*σ*_e_)-induced rupture of GUVs in the presence of different osmotic pressures (*Π*). (a) A schematic diagram of the experimental set-up for the constant-electric-tension (*σ*_e_)-induced rupture of GUVs under osmotic pressure. (b) The time course of the fraction of intact 46%DOPG/39%DOPC/15%chol-GUVs at *σ*_e_ tensions of 6.5, 6.0, and 5.0 mN m^−1^. The solid lines show the best fit curves using eqn (4). (c) The *σ*_e_-dependent *k*_r_ values for 46%DOPG/39%DOPC/15%chol-GUVs under osmolarity differences of Δ*C*^0^ = 0 mOsm L^−1^ (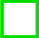), Δ*C*^0^ = 15 mOsm L^−1^ (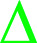), and Δ*C*^0^ = 19 mOsm L^−1^ (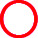). The average *k*_r_ values with standard deviations at various *σ*_e_ values were determined using three independent experiments, each of which was conducted using 18−24 GUVs for each *σ*_e_. The solid (green, cyan, and red) lines are the best fit curves of eqn (5) using *Γ* = 12.9 pN, *B* = 2.14 mN m^−1^, and *A*_F_ = 8.4 × 10^5^ m^2^ s^−1^ J^−1^. The cyan line and the red line correspond to the theoretical eqn (5) using *σ*_t_ = *σ*_c_ + 2.3 mN m^−1^ and *σ*_t_ = *σ*_e_ + 4.0 mN m^−1^, respectively. (d) The Δ*C*^0^-dependent membrane tension (*σ*_oseq_) (experimental and theoretical) at swelling equilibrium. (e) The *σ*_e_-dependent *k*_r_ values for 40%DOPG/60%DOPC-GUVs at Δ*C*^0^ = 0 mOsm L^−1^ (□), Δ*C*^0^ = 13 mOsm L^−1^ (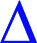), and Δ*C*^0^ = 17 mOsm L^−1^ (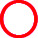). Average *k*_r_ values with standard deviations at various *σ*_e_ values were determined from three independent experiments, each of which was conducted using 15−24 GUVs for each *σ*_e_. The solid (black, blue, and red) lines are the best fit curves of eqn (5) using *Γ* = 12.1 pN, *B* = 1.76 mN m^−1^, and *A*_F_ = 8.8 × 10^6^ m^2^ s^−1^ J^−1^. The blue line and the red line correspond to the theoretical eqn (5) using *σ*_t_ = *σ*_e_ + 2.1 mN m^−1^ and *σ*_t_ = *σ*_e_ + 3.5 mN m^−1^, respectively. (f) The Δ*C*^0^-dependent membrane tensions (*σ*_oseq_) (experimental and theoretical) at swelling equilibrium. The images in (b–f) have been adapted from ref. 11 with permission from PLOS, copyright 2021.

An illustration of the application of a concentration gradient (Δ*C*^0^) to GUVs under an osmotic pressure *Π* is provided in Fig. 14(a). Fig. 14(b) shows the time course of *P*_intact_ (*t*) for 46%DOPG/39%DOPC/15%chol-GUVs at *σ*_e_ = 6.5, 6.0 and 5.0 mN m^−1^ at Δ*C*^0^ = 15 mOsm L^−1^ [a detailed description of the calculation of *P*_intact_ (*t*) is provided in Section 3.1]. The data was fitted well using eqn (4), and *k*_r_ values of 0.9 × 10^−1^, 3.7 × 10^−2^ and 0.9 × 10^−2^ s^−1^ were obtained for *σ*_e_ = 6.5, 6.0 and 5.0 mN m^−1^, respectively. Fig. 14(c) shows the *σ*_e_-dependent *k*_r_ values for Δ*C*^0^ = 0, 15 and 19 mOsm L^−1^.

The membrane tension (*σ*_oseq_) induced by the osmotic pressure was then estimated at the swelling equilibrium. The total membrane tension was *σ*_t_ = *σ*_e_ + *σ*_oseq_, in which *σ*_e_ is due to the electric tension and *σ*_oseq_ is due to the osmotic pressure. The rate constant of rupture was determined for *σ*_t_. The shifting of the *k*_r_*vs. σ*_e_ curve from the right to the left side with increasing Δ*C*^0^ corresponds to *σ*_oseq_ (Fig. 14(c)). The value of *σ*_oseq_ was estimated experimentally by subtracting the value of *σ*_e_ at *Π* (to induce a specific *k*_r_) from the value of *σ*_e_ at Δ*C*^0^ = 0 (to induce the same *k*_r_). In the absence of osmotic pressure (*i.e.*, *σ*_oseq_ = 0), *σ*_t_ = *σ*_e_. The experimental *k*_p_*vs. σ*_e_ data for 46%DOPG/39%DOPC/15%chol-GUVs at Δ*C*^0^ = 0 was fitted using eqn (5) (where *k*_p_ is the rate constant of tension-induced pore formation). For Δ*C*^0^ = 15 mOsm L^−1^, the value of *k*_r_ was 1.1 × 10^−1^ s^−1^ at *σ*_e_ = 6.5 mN m; using eqn (5), the same *k*_r_ was induced at *σ*_e_ = 9.0 mN m^−1^ for Δ*C*^0^ = 0.

By subtracting the above tensions, the experimental membrane tension at swelling equilibrium *σ*_osexp_ was determined to be 2.5 mN m^−1^. The value of *σ*_osexp_ was also determined for other values of *k*_r_ (Fig. 14(c)). The average value of *σ*_osexp_ was determined to be 2.3 ± 0.2 mN m^−1^. Using the relation *σ*_t_ = *σ*_e_ + 2.3 mN m^−1^ and the same parameters as for Δ*C*^0^ = 0 mOsm L^−1^, the experimental data for Δ*C*^0^ = 15 mOsm L^−1^ was fitted using eqn (5) (Fig. 14(c)). Similarly, for Δ*C*^0^ = 19 mOsm L^−1^, the experimental data was fitted using *σ*_t_ = *σ*_c_ + 4.0 mN m^−1^ (Fig. 14(c)). The values of the theoretical membrane tension, *σ*_osthe_, were calculated for 15 and 19 mOsm L^−1^ using eqn (11). The Δ*C*^0^-dependent membrane tension for the 46%DOPG/39%DOPC/15%chol-GUVs is shown in Fig. 14(d). Fig. 14(e) shows the *σ*_e_-dependent *k*_r_ values for various Δ*C*^0^ values for the 40%DOPG/60%DOPC-GUVs. In a similar way to that described above, the data were fitted using *σ*_t_ = *σ*_e_ + 2.1 for Δ*C*^0^ = 13 mOsm L^−1^ and *σ*_t_ = *σ*_e_ + 3.5 mN m^−1^ for Δ*C*^0^ = 17 mOsm L^−1^. The Δ*C*^0^-dependent membrane tension for the 40%DOPG/60%DOPC-GUVs is shown in Fig. 14(f). The values of the experimental and theoretical membrane tension under various conditions are provided in Table 2.

**Table 2:** Estimation of membrane tension under various concentration gradients

Membrane composition (% indicates mol%)	Measurement technique	Concentration gradient, Δ*C*^0^ (mOsm L^−1^ or mM)	Experimental membrane tension, *σ*_osexp_ (mN m^−1^)	Theoretical membrane tension, *σ*_osthe_ (mN m^−1^)
46%DOPG/39%DOPC/15%chol	Electroporation	15 mOsm L^−1^	2.3 ± 0.2	4.1 ± 1.4 (ref. 11)
46%DOPG/39%DOPC/15%chol	Electroporation	19 mOsm L^−1^	4.0 ± 0.3	5.2 ± 1.3 (ref. 11)
40%DOPG/60%DOPC	Electroporation	13 mOsm L^−1^	2.1 ± 0.3	3.3 ± 1.2 (ref. 11)
40%DOPG/60%DOPC	Micropipette	12 mOsm L^−1^	2.6 ± 0.2	3.0 ± 1.2 (ref. 10)
40%DOPG/60%DOPC	Electroporation	17 mOsm L^−1^	3.5 ± 0.1	4.3 ± 2.3 (ref. 11)
40%DOPG/60%DOPC	Micropipette	17 mOsm L^−1^	3.4 ± 0.2	4.2 ± 1.3 (ref. 10)
DOPC	Micropipette	1.9 mM	2.6 ± 0.1	2.6 ± 0.7 (ref. 9)
DOPC	Micropipette	2.8 mM	3.8 ± 0.1	3.9 ± 0.7 (ref. 9)

### 3.9. Estimation of membrane tension under different osmotic pressures using constant-mechanical-tension-induced rupture in GUVs

**Fig. 15:**
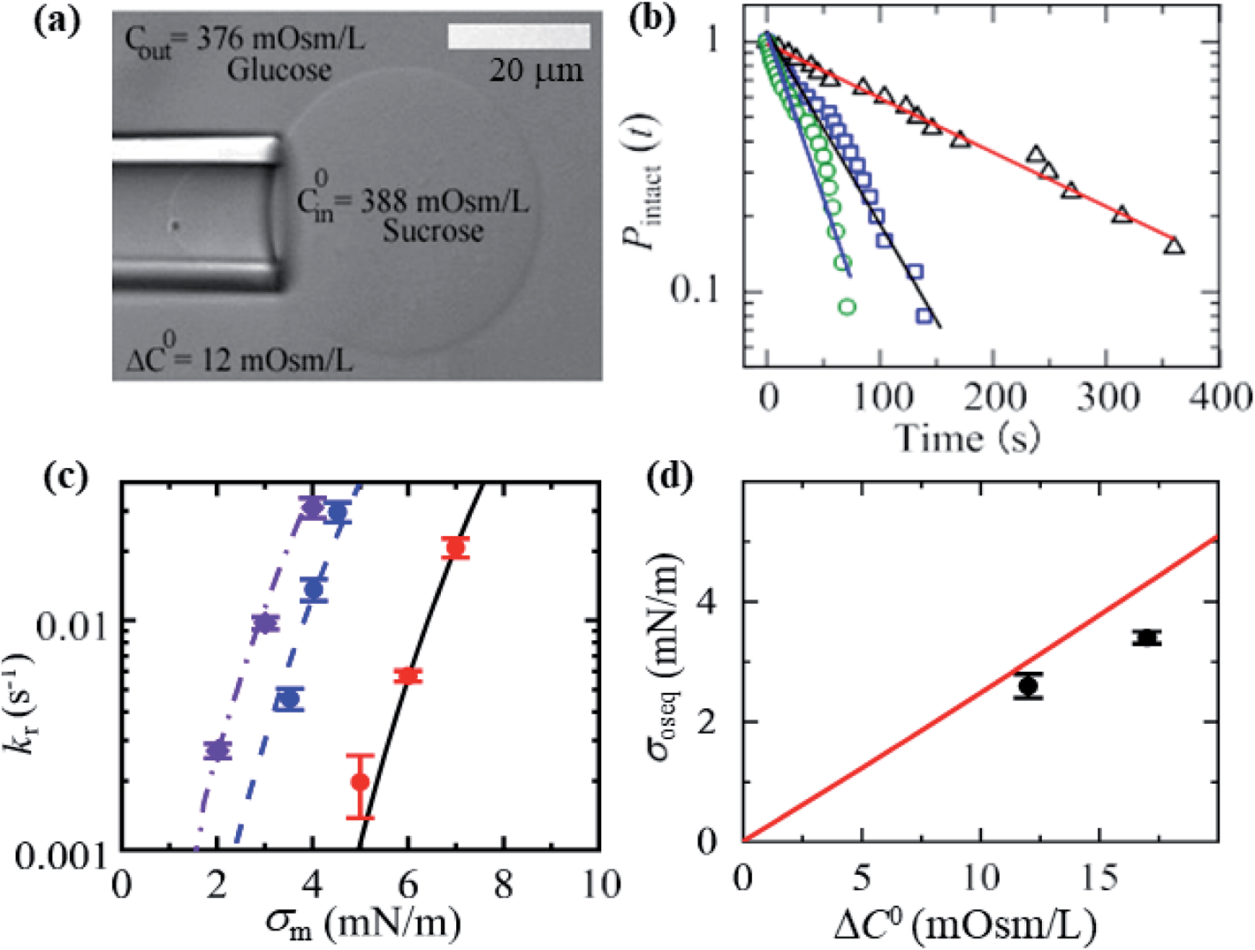
Constant-mechanical-tension (*σ*_m_)-induced rupture of 40%DOPG/60%DOPC-GUVs in the presence of various osmotic pressures (*Π*). (a) A differential interference contrast microscopic image of a GUV aspirated using a micropipette at *σ*_m_ = 3.5 mN m^−1^ and Δ*C*^0^ = 12 mOsm L^−1^. (b) The time course of *P*_intact_ (*t*) at tensions *σ*_m_ of 3.5 (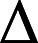), 4.0 (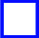), and 4.5 (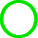) mN m^−1^ under Δ*C*^0^ = 12 mOsm L^−1^. The number of GUVs examined was 20–25 in each experiment. The solid lines represent the best fit curves using eqn (4). (c) The *σ*_m_-dependent *k*_r_ values in the presence of Δ*C*^0^ = 17 mOsm L^−1^ (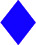), Δ*C*^0^ = 12 mOsm L^−1^ (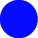), and Δ*C*^0^ = 0 mOsm L^−1^ (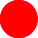). The solid lines are the best fit curves of eqn (5) using *Γ* = 11.4 pN, *B* = 2.6 mN m^−1^, and *D*_r_ = 165 nm^2^ s^−1^. The purple dash–dotted line and the blue dashed line correspond to eqn (5) using *σ*_t_ = *σ*_m_ + 3.4 mN m^−1^ and *σ*_t_ = *σ*_m_ + 2.6 mN m^−1^, respectively. (d) Concentration-gradient (Δ*C*^0^)-dependent membrane tension (*σ*_oseq_) at swelling equilibrium. Mean values with standard deviations are shown. The red line indicates the theoretical curve of eqn (11). The images in a–d have been adapted from ref. 10 with permission from American Chemical Society, copyright 2020.

In this section, we describe the constant-mechanical-tension (*σ*_m_)-induced rupture of 40%DOPG/60%DOPC-GUVs under various osmotic pressures (*Π*) and estimation of their corresponding membrane tension (*σ*_oseq_) at swelling equilibrium. The *σ*_m_-dependent rate constant (*k*_r_) of rupture was determined as described in Section 3.2. To apply an osmotic pressure to the GUVs, first, the GUVs were transferred into a buffer with a lower osmolarity than that of the GUV lumen in a microchamber (Fig. 15(a)). The osmolarity of the sucrose inside the GUVs in the initial stage was *C*^0^_in_ = 388 mOsm L^−1^. When this GUV suspension was mixed with a buffer containing 80 mM glucose in a microchamber in a volume ratio of 1 : 14, the outside osmolarity of the GUVs was *C*_out_ = 376 mOsm L^−1^, and hence Δ*C*^0^ = 12 mOsm L^−1^. The GUV suspension was incubated in the microchamber for about 5 min to reach the swelling equilibrium. The time course of *P*_intact_ (*t*) for Δ*C*^0^ = 12 mOsm L^−1^ was investigated at *σ*_m_ = 3.5, 4.0 and 4.5 mN m^−1^, and the *k*_r_ value for each tension was then obtained (Fig. 15(b)). Fig. 15(c) shows the *σ*_m_-dependent *k*_r_ for the 40%DOPG/60%DOPC-GUVs in the presence of Δ*C*^0^ = 0, 12 and 17 mOsm L^−1^ (Fig. 15(c)), which indicated that as Δ*C*^0^ increased, lower tension was required to obtain a similar *k*_r_ value. The total membrane tension was *σ*_t_ = *σ*_m_ + *σ*_oseq_, where *σ*_m_ was due to mechanical tension and *σ*_oseq_ was due to osmotic pressure. The value of *σ*_oseq_ was obtained by subtracting the value of *σ*_m_ under a given *Π* (to induce a specific *k*_r_) from the value of *σ*_m_ with *Π* = 0 (to induce the same *k*_r_). If *σ*_m_ = *σ*_t_, *σ*_oseq_ = 0. The experimental *k*_r_*vs. σ*_m_ data with Δ*C*^0^ = 0 mOsm L^−1^ was fitted well by eqn (5) (Fig. 15(c)). Using the relation *σ*_t_ = *σ*_m_ + 2.6 mN m^−1^ and the same parameters as used for Δ*C*^0^ = 0 mOsm L^−1^, the experimental data for Δ*C*^0^ = 12 mOsm L^−1^ was fitted using eqn (5) (Fig. 15(c)). Similar steps were followed for Δ*C*^0^ = 17 mOsm L^−1^. Fig. 15(d) shows that the *σ*_oseq_ increased with Δ*C*^0^. The red solid line shows the theoretical curve of eqn (11). The experimental and theoretical membrane tension values due to various osmotic pressures for different membrane compositions are provided in Table 2.

The literature presented on osmotic pressure clearly indicates that lipid vesicles become weak in the presence of osmotic pressure. Higher osmotic pressure creates pores in the membranes of GUVs even in the absence of external tension.^9,10^ To prevent osmotic-pressure-induced cell death, cells change their biophysical structure by incorporating mechanosensitive channels in membranes^3^ during their development. The gates of the mechanosensitive channels open when the plasma membranes are stretched by osmotic pressure,^1,2^ and consequently, the possibility of cell death under osmotic pressure is reduced.

### 3.10. Critical tension of rupture in GUVs using the electroporation technique

**Fig. 16:**
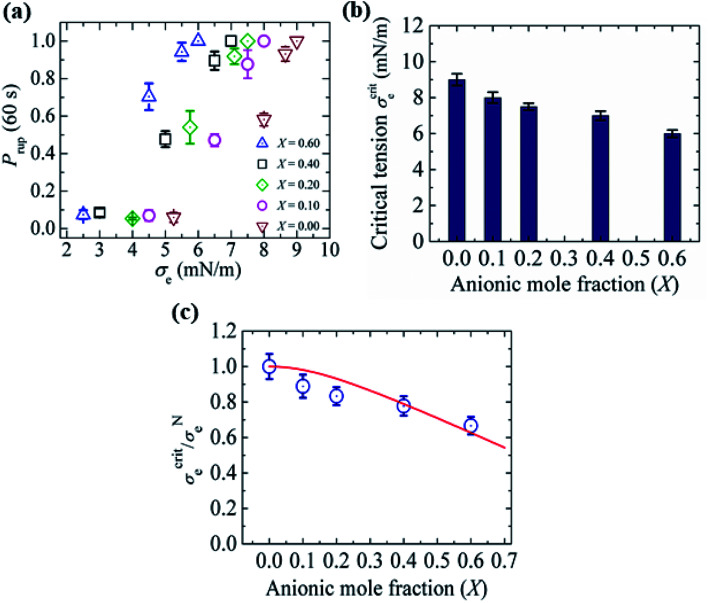
The critical tension of rupture of DOPG/DOPC-GUVs using the electroporation technique. (a) The constant-electric-tension (*σ*_e_)-dependent probability of rupture of the GUVs. (b) Bar chart of the anionic-lipid-fraction-dependent critical tension of rupture. (c) The normalized critical tension of rupture of the GUVs. The solid red line in (c) shows the best fit curve of eqn (15) using *K* = 0.35. The images in a–c have been adapted from ref. 117 with permission from European Biophysical Societies' Association, copyright 2020.

In this section, the critical tension of rupture of GUVs with various anionic lipid mole fractions (*X*) is described, which is used for measuring the strength of the lipid bilayer.^25^ In the electroporation technique, the critical tension (*σ*^crit^_e_) of rupture was defined as the minimum tension required for the macroscopic pore formation without any further free energy barrier.^117^ In this case, the critical tension was obtained when the probability of rupture by a time of 60 s, *P*_rup_ (60 s), was equal to 1.0. The calculation of *P*_rup_ (60 s) for various electric tensions was described in Section 3.1. When *P*_rup_ (60 s) = 1.0, the critical tension *σ*^crit^_e _ was 7.0 mN m^−1^ for *X* = 0.40 (Fig. 16(a)). Similarly, *σ*^crit^_e_ was obtained for various *X* values. As the value of *X* was increased from 0.0 to 0.60, the value of *σ*^crit^_e_ decreased from 9.0 ± 0.3 to 6.0 ± 0.2 mN m^−1^. These results indicated that the mechanical stability of the membranes greatly decreased with increasing anionic lipid content in the membranes. The bar chart of the *X*-dependent critical tension is presented in Fig. 16(b). The *X*-dependent normalized critical tension is shown in Fig. 16(c).

According to Gouy–Chapman–Stern theory, surface charge is determined by the fraction of anionic lipids (*X*) in membranes and the ion-lipid binding (*B*_in_). After binding, the effective anionic lipid fraction is expressed using a Langmuir-type equilibrium as follows:^118,119^12
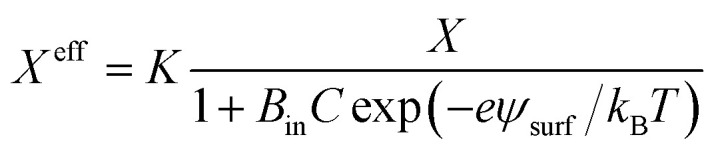
Here, *K* is a fitting parameter and *C* is the salt concentration. The surface potential (*ψ*_surf_) of the charged membrane is estimated by the Graham equation as follows:^82^13
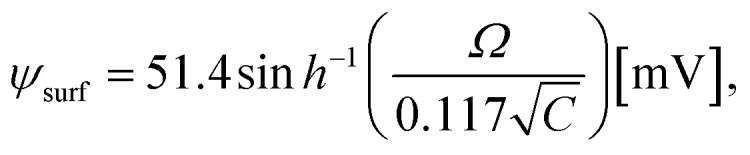
where *Ω* is the surface charge density. The critical tension of rupture can be expressed as follows:14
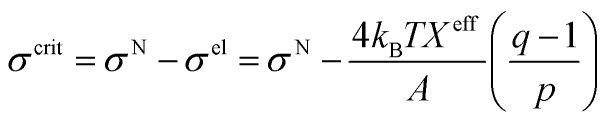
Here, *σ*^el^ is the effective electrostatic tension in the membranes and *σ*^N^ is the critical tension for a neutral membrane. After normalization, eqn (14) can be written as follows:15
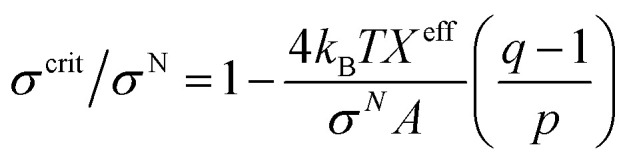


It should be noted that this expression can be used for *σ*_e_ and *σ*_m_ as well. The experimental data (Fig. 16(c)) was fitted using eqn (15), and the obtained first-order binding constant between ions in the bathing solution and the anionic lipid membrane, *B*_in_, was calculated to be 0.75 M^−1^ for PG^−^:Na^+^.

### 3.11. Critical tension of rupture in GUVs using the micropipette aspiration technique

**Fig. 17:**
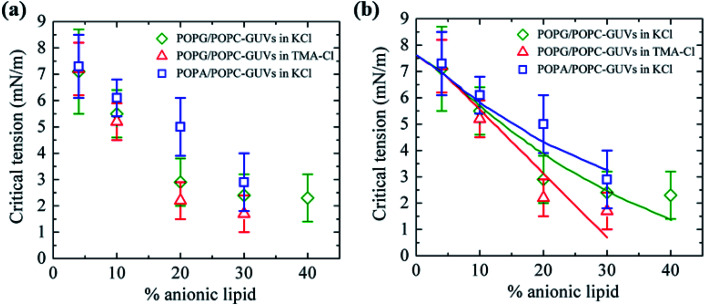
The anionic-lipid-dependent critical tension for the rupture of GUVs using the micropipette technique. (a) POPG/POPC in KCl (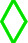), POPG/POPC in TMA-Cl (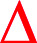), and POPA/POPC-GUVs in KCl (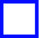). (b) The experimental anionic-lipid-dependent critical tension data for POPG/POPC in KCl (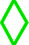), POPG/POPC in TMA-Cl (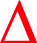), and POPA/POPC-GUVs in KCl (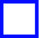), fitted using eqn (14). The images in a and b have been adapted from ref. 119 with permission from The Biophysical Society (published by Elsevier Inc), copyright 2002.

This section describes the critical tension of rupture using the micropipette aspiration technique. With increasing mechanical tension (*σ*_m_), the fractional change in the area of the GUV increases, which provides the area compressibility modulus of the membranes. When the tension exceeds a critical level, the GUV ruptures. In this case, phosphatidylglycerol (POPG)/phosphatidylcholine (POPC)-GUVs were prepared in potassium chloride (KCl), POPG/POPC-GUVs were prepared in tetramethylammonium chloride (TMA) and palmitoyloleoylphosphatidic acid (POPA)/POPG-GUVs were prepared in KCl by varying the anionic lipid fraction from 0.04 to 0.40. The anionic-lipid-dependent critical tension for different lipid compositions is presented in Fig. 17(a). The values of the binding constant (*B*_in_) for PA^−^:K^+^, PG^−^:K^+^ and PG^−^:TMA^+^ obtained from the fitting of experimental data were 0.80, 0.40 and 0.0 M^−1^, respectively (Fig. 17(b)) with eqn (14). In the case of DOPG/DOPC-GUVs in PIPES buffer, the value of *B*_in_ was 0.75 M^−1^, as described in Section 3.10. Moreover, previous works reported *B*_in_ values of 0.0–1.10 M^−1^ for different types of salts.^105,118,120^ The estimated values of *B*_in_ obtained using the aspiration technique are similar to those for the electroporation technique.

## 4. Biological and technological aspects

External stimulation, such as the application of mechanical stress (*i.e.*, tension) at the mechanosensitive ion channels (MSCs) (nonspecialized cells) using the patch pipette technique activated the mechanosensitivity of MSCs from their resting condition.^3^ Such MSCs are present in the membranes of organisms of the bacteria, archaea and eukaryote domains. In addition, mechanical tension can modulate different channels that have the same properties as MSCs, but lower energies. As an example, the kinetics of voltage-sensitive channels change by several orders of magnitude in response to mechanical stress. MSCs are not only strongly gated by voltage, but also effectively gated by amphipathic ligands such as unsaturated fatty acids and general anesthetics.^121–123^ Additionally, tension occurs in the case of lipid–glass adhesion, which is calculated by measuring the angle at which the patch membrane contacts the glass.^124^ The role of the cell adhesion molecule *Neuroligin 2* at the *Drosophila* neuromuscular junction for synaptic development and functioning has been characterized. *Drosophila neuroligin 2* can colocalize and hence bind to *Drosophila* neurexin.^125^ The role of a noncoding RNA called the human 7SK small nuclear RNA in regulating eukaryotic transcription has been investigated.^126^ Signaling pathways, stress, ultraviolet light and other external cues lead to the release of positive transcription elongation factor *b*. The membrane tension due to the swelling of a ‘single cell’ in the presence of an osmotic gradient provided important information for its potential applications in biological studies and clinical practice. In this case, a single cell was trapped using a microfluidic device and the swelling dynamics were evaluated; changes in the mechanical properties of cells due to cytoskeleton disruption could be detected.^127^

Membrane stretching using the micropipette aspiration technique activated the antimicrobial peptide, Magainin 2, inducing pore formation in GUVs.^12,13^ Here, the rate constant of the Magainin-2-induced pore formation increased with increasing external membrane tension. Additionally, the entry of the cell-penetrating peptide Transportan 10 into single GUVs without pore formation is modulated by the constant tension.^21,128^ The transbilayer movement of lipids was observed in the presence of a constant external tension.^129^ The penetration of nanoparticles into a ‘single GUV’ was greatly influenced by the lateral membrane tension;^130^ this tension was generated by an osmotic gradient. This type of penetration of particles and peptides into vesicles through the lipid bilayer is potentially useful for controlling living cells, such as for gene delivery,^131,132^ local heating,^133^ and the visualization of proteins.^134^ Based on these discussions, it can be concluded that the constant membrane tension plays an important role in various biophysical, biochemical and biological processes.

Research into constant electric tension induced by a pulsed electric field, such as electro-kinetics phenomena, has been conducted in biology and biotechnology.^135^ Promising applications of electroporation in biotechnology include genetic transformation (*i.e.*, the entrance of anticancer drugs and nucleic acids into the targeted cells and tissues), microorganism inactivation, extraction of intracellular compounds from microorganisms and tissues, and biomass drying.^136–138^ Electroporation technology has been successfully used for tissue decellularization to produce tissue- and organ-derived scaffolds by removing the cellular contents while preserving the important structural and biochemical features to support cell growth.^139^ Several surgical applications using this technology are now under study or even in clinical trial, *e.g.*, ablation of hepatocarcinomas, ablation of prostate tumors, treatment of atrial fibrillation and treatment of vascular issues such as restenosis and atherosclerotic processes.^140^ This technology has also been implemented in food preservation systems, food processing and biorefinery.^141,142^ Localized prostate, kidney, lymph, and lung cancer treatments, along with liver tumor ablation, by the electroporation technique are well studied.^143,144^ Therefore, the technological aspects of constant electric tension due to electroporation can be obtained in the fields of biomedical, bioengineering, and biotechnological applications.

## 5. Concluding remarks

This review describes the kinetics of the constant-electric- and mechanical-tension-induced rupture of GUVs. Emphasis is placed on outlining the effects of surface charge, salt concentration in the buffer, cholesterol content in the membrane, and lipid composition. The changes in the mechanical stability and line tension under those conditions are discussed. Interestingly, these results are very similar for both techniques. The mechanism of rupture is explained based on the classical theory of pore formation, although there is scope for further investigation of the detailed mechanism. The membrane tension varies with osmotic pressure, as described for different membrane compositions. The critical tension of vesicle rupture measures the strength of the lipid bilayer. These observations provide understanding of the effects of electric fields, mechanical stress, and osmotic pressure on the membranes of vesicles and cells.

It has been reported that sugar concentration changes the bending rigidity of membranes,^145,146^ as sugar molecules interact strongly with lipid bilayers and act as an additive. However, the GUVs were prepared in the absence of a membrane potential, although real cells maintain a membrane potential of approximately −70 mV under resting conditions.^147^ Very recently, it was reported that the membrane potential influences the kinetics of peptide-induced pore formation in lipid vesicles.^23,148,149^ The mechanical properties of neutral GUVs, such as the bending modulus of the saturated/monounsaturated chain, increased with chain length. However, for polyunsaturated chains (*i.e.*, two or more *cis* double bonds in the chain), the bending modulus dropped significantly, which was explained by the decrease of the membrane thickness, *i.e.*, the distance between the peak-to-peak headgroups of the lipid.^102^ Therefore, there is scope to investigate the constant-electric- and -mechanical-tension-induced rupture of GUVs and correspondingly estimate the line tension upon varying the sugar concentration, membrane potential, and polyunsaturated lipids. The study of GUV rupture using various types of salts is still missing. In addition, there is scope for investigating the effects of the frequency of the IRE signal on the kinetics of rupture under constant electric tension.

## Author contribution

MASK, MKA and MA designed the review. MKA and MA arranged the graphs and data. MASK and ZBM wrote the paper.

## Conflicts of interest

The authors declare no conflicts of interest.

## Supplementary Material
